# Supplementary material for: Multimodal confined water dynamics in reverse osmosis polyamide membranes
Source: Nat Commun. 2022 May 19;13:2809. doi: 10.1038/s41467-022-30555-6 (PMC9120036; doi:10.1038/s41467-022-30555-6)
Supplement: Supplementary file 1 — Supplementary Information [file 41467_2022_30555_MOESM1_ESM.pdf]

Supplementary Information for

Multimodal confined water dynamics in reverse osmosis polyamide membranes

Fabrizia Foglia[1,†*], Bernhard Frick[2], Manuela Nania[1], Andrew G. Livingston[1,‡], and João T. Cabral[1*]
[1] Department of Chemical Engineering, Imperial College London, London SW7 2AZ, United Kingdom;
[2] Institut Laue Langevin, 71 avenue des Martyrs - CS 20156 - 38042 Grenoble CEDEX 9, France
[†] Present address: Chemistry Department, Christopher Ingold Laboratories, University College London, London WC1H 0AJ, United Kingdom, Email: f.foglia@ucl.ac.uk,
[‡] Present address: School of Engineering and Materials Science, Queen Mary University of London, Mile End Road, London E1 4NS, United Kingdom.
E-mail: j.cabral@imperial.ac.uk

Index

Supplementary Note 1. Differential Scanning Calorimetry (DSC)

Supplementary Note 2. Neutron Scattering

Supplementary Note 3. Quasielastic neutron scattering (QENS)

Supplementary Note 4. Model for the dry polymer

Supplementary Note 5. Model for the hydrated polymer in D_2O

Supplementary Note 6. Model for the hydrated polymer in H_2O

Supplementary Note 7. Water uptake and proton fraction

Supplementary Note 8. Relationship between multi-modal diffusion and membrane permeance

Supplementary Note 1. *Differential Scanning Calorimetry (DSC)*

Differential scanning calorimetry (DSC) experiments were carried out using a DSC131, Setaram, and approximately 30 mg of sample in each repetition. To ensure statistical reproducibility, each experiment was repeated three times. Dry samples were loaded in aluminium crucibles with a capacity of 30 mm^3 and subjected to a temperature profile shown in Supplementary Fig. 1a. The samples were first stabilised at room temperature (isotherm-1), then cooled to 170 K (T_c-ramp-1), equilibrated for 5 min (isotherm-2) and then heated to 460 K (T_c-ramp-2) and held isothermally for 5 min (isotherm-3); the cycle was repeated and the sample returned to room temperature. Heating and cooling ramps were recorded at 10 $Kmin^{-1}$. As shown in Supplementary Fig. 1b, the polymer glass transition (T_g) is centered around 400 K, as expected for fully aromatic PA samples.

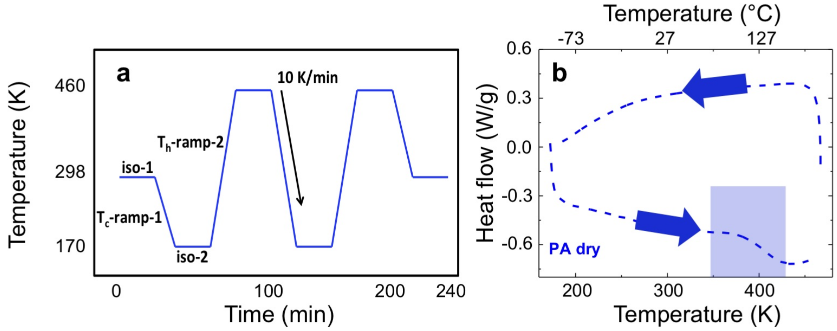

Supplementary Figure 1: a) Differential scanning calorimetry (DSC) temperature profile employed to measure the PA polymer in its dry state. b) Heat flow profile, showing the direction of cooling and heating ramps. Highlighted area indicates $T_g \simeq 400$ K. Source data are provided as a Source Data file.

Supplementary Note 2. *Neutron spectroscopy*

The combination of time-of-flight (TOF, IN5) and a high-resolution backscattering (BS, IN16B), illustrated in Supplementary Fig. 2, allows the examination of the mechanisms, geometry and frequency of translational diffusive water motions at the Å-scale in a broad time window, from ps to tens of ns, and thus both short- and long-correlation lengths. Data normalization and analysis were carried out using Large Array Manipulation Program (LAMP) [1] and Microcal Orign 2018b; the raw data were normalised to the neutron flux, as well as detector efficiency fluctuations by direct comparison with the purely incoherent signal from a vanadium reference sample. The double differential cross section was then converted into the corresponding dynamic structure factor S(Q,ω). Additional normalization steps, such as removing the signal associated to the empty can, and data analysis were carried out directly on the S(Q,ω) spectra using either LAMP or Microcal Origin 2018b. Each Q-slice was analyzed using a built-in least squares algorithm accounting for the instrumental energy resolution along with up to two Lorentzian functions, related to the polymer segmental relaxation and water translational diffusion.

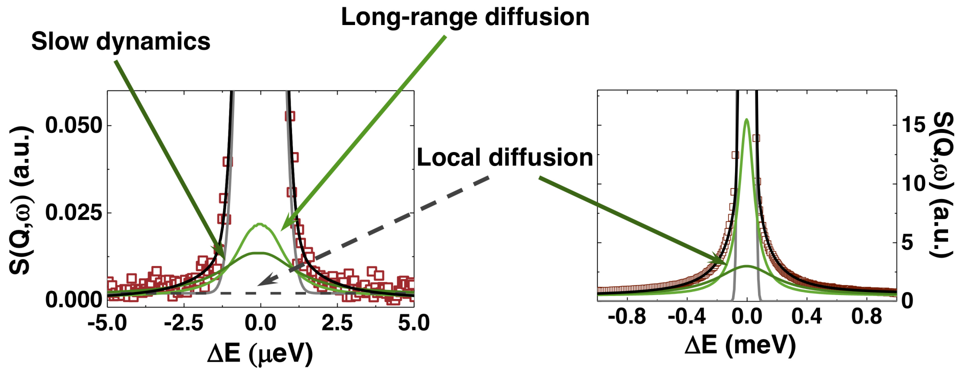

Supplementary Figure 2: Comparison between scattering profiles acquired at two instrumental resolutions, namely 1 μeV in BS (IN16B) and 45 μeV in TOF (IN5), illustrating the different contributions of dynamic structure factors in both windows. The combination of two energy resolutions and four sample series (dry PA, D_2O vapor hydrated PA, and H_2O vapor hydrated PA, and liquid immersed) enables the elucidation and decoupling of the various relaxation and diffusive processes present in PA membranes used in reverse osmosis (RO) water desalination.

Supplementary Note 3. *Quasielastic neutron scattering (QENS)*

Fixed Window Scan (FWS) for dry and hydrated PA films were obtained by measuring the scattering signal at either ΔE $=$ 0 (EFWS; Supplementary Fig. 3a-b) or $=$ 2 μeV (IFWS; Supplementary Fig. 3c-d), as a function of temperature, from 2 to 380 K, and heating rate of 0.13 Kmin^{-1}, using backscattering spectrometer IN16B. Elastic Fixed Window Scans (EFWS) provide an effective means to locate temperature window when the relaxation dynamics of the system enters the time (or energy) window of the neutron spectrometer, and thus select measurement QENS temperatures, generally where a change in slope is visible on the elastic scan. Further, the temperature dependence of the mean-square displacement (msd) of hydrogen (H) atoms can be computed according to:

$$\frac{I_{inc-elastic}(Q,T)}{I_{inc-elastic}(Q,T_{min})} = exp\left(-\frac{1}{3}Q^2\left(\langle u^2\rangle - \langle u^2\rangle_{T_{min}}\right)\right) \quad \text{(S1)}$$

assuming that oscillations are harmonic and isotropic. The msd is generally computed from the slope of the logarithmic of Eq. (S1) above, as shown in Supplementary Fig. 3g-j, taking $ln(I_{el}(T)/Iel(T_{min}))\ vs\ Q^2$ and T_{min} is sufficiently low (2 K in our case). We have computed the msd in two different ways, considering either (i) a small Q-range (Supplementary Fig. 3g-h) or (ii) the full Q-range (Supplementary Fig. 3i-j). The difference between these two reflects the reduced linearity at high-Q and -T due to the increased anharmonicity of the oscillations. The latter is also the reason why msd is computed from the slope only at low temperatures (T $\leq$ 100 K; Fig. 1a, main paper), where the Debye-Waller approximation holds, beyond which we refer to an apparent Debye-Waller factor. These estimates are comparable with molecular dynamics simulations (in the temperature range between 250 to 325 K; supplementary Fig. 3k) discussed below.

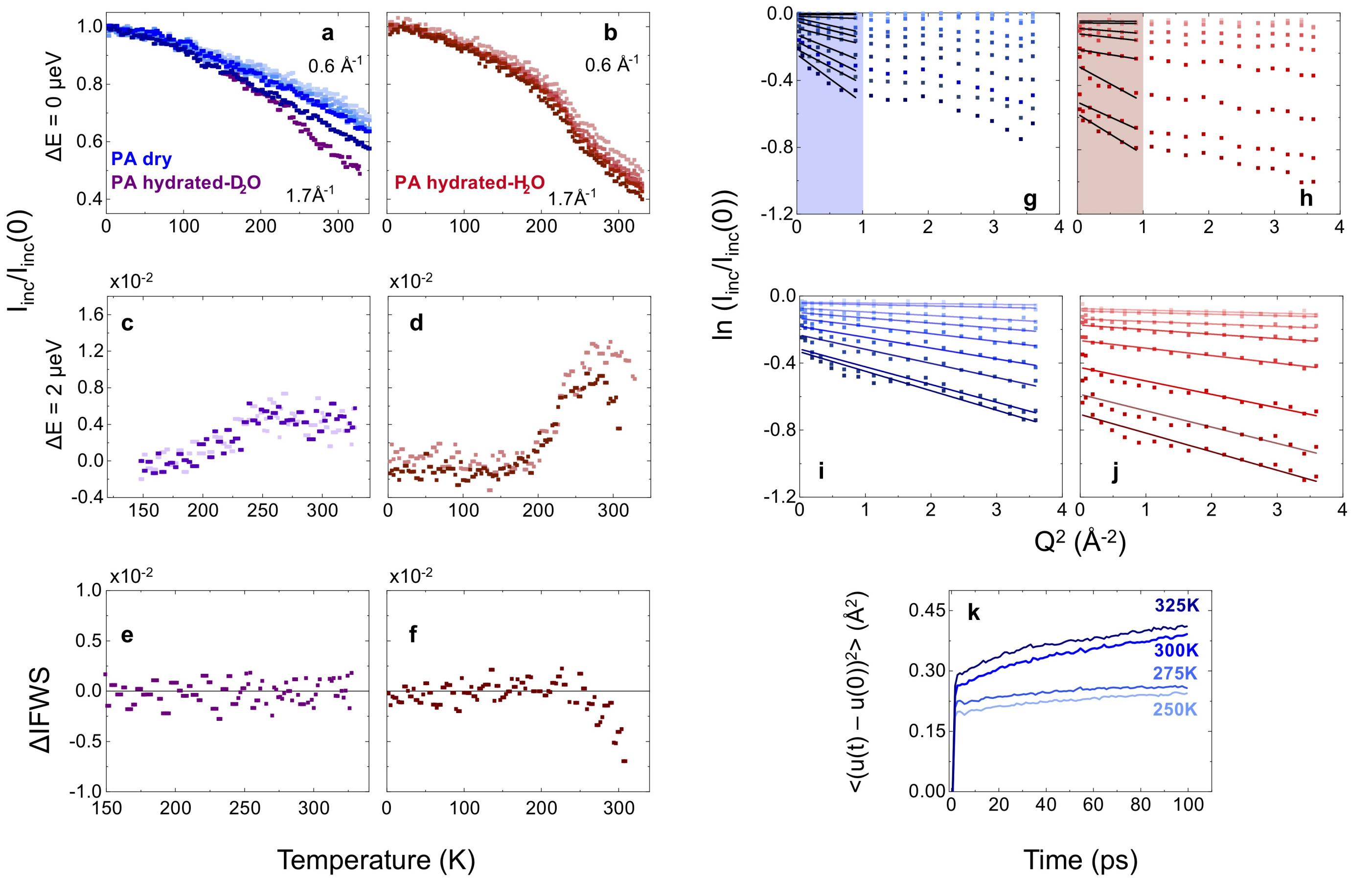

Supplementary Figure 3: Elastic and inelastic fixed window scan (shown at $Q = 0.6$, 0.9, 1.2, 1.5 Å^{-1}) for PA membrane sample in its dry (blue markers), D_2O-hydrated (purple markers) and H_2O-hydrated (red markers) state. a-b) EFWS and (c-d) IFWS. e-f)ΔIFWS to highlight the peak shift in the case of H_2O-hydrated. Panels (g-j) report the fits used to evaluate the mean-square displacement ($\langle u^2 \rangle$) with a restricted (g-h) and full (i-j) Q range. k) Time variation of the mean square displacement (msd) for cross-linked PA membrane in its dry state between 250 and 325 K obtained from MD simulations. Source data are provided as a Source Data file.

In addition, Inelastic Fixed Window Scans (IFWS) were also recorded for the same set of samples, as shown in Supplementary Fig. 3c-f. The temperature scan probes the variation of the polymer chain mobility and associated relaxation time, inducing a change in the inelastic signal, which reaches a maximum when the QENS broadening matches ω_{off} [2]. Therefore, IFWS can further discriminate between local (for instance rotational) and diffusive motions, for which τ (and thus T_{max}) is Q-independent or Q-dependent, respectively as shown in Fig. 1b-c of the main paper, and Supplementary Fig. 3c-d below. Following Frick and co-workers [2]:

$$I^{IFWS}_{\omega_{off}}(T) \propto \frac{B}{\pi}(1 - A_0(Q)) \frac{\tau(T)}{1 + \omega^2_{\omega_{off}} \tau(T)^2} \quad \text{(S2)}$$

$$\tau(T) = \tau_0 exp\left(-\frac{E_a}{KT}\right) \quad \text{(S3)}$$

$$T_{max} = \frac{E_a/KT}{ln\,(1/\omega_{off}\tau_0)} \quad \text{(S4)}$$

where B is a constant which accounts for the resolution function, ω_{off} is the energy offset, τ is the relaxation time, τ_0 is the high τ-limit of the relaxation time, A_0 is the elastic incoherent structure factor (EISF), K is the Boltzmann

constant and E_a is the activation energy. In our case the diffusive motions of confined water are clearly visible in the H_2O hydrated PA membrane (Fig. 1b, main paper) and are not observed when the membrane is hydrated with D_2O vapor instead (Fig. 1c, main paper). The latter characterise the polymer segmental motions in the swollen state, whose contribution is then fixed to evaluate the water dynamics with higher precision.

In incoherent quasi-elastic neutron scattering (QENS) [3, 2], the measured signal provides spatio-temporal correlations of single H atoms, which dominate the scattering cross-section, whose motion can be described terms of vibrational, translational and rotational components convoluted in reciprocal space

$$S_{inc}(Q,\omega) = S_{vib}(Q,\omega) \otimes S_{rot}(Q,\omega) \otimes S_{trans}(Q,\omega) \tag{S5}$$

In the case of isotropic, harmonic vibrations, the first term can be written as S_{vib} $(Q,\omega) = e^{-Q^2<u^2>/3}$ accounting for proton delocalisation as timescales much faster than the instrumental window. Further, immobile protons (or moving slower than the instrumental resolution) give rise an elastic scattering ($\delta(\omega)$) term in the equation above, while fast dynamics (outside the experimental window) contribute as a flat background, $B(Q)$. The dynamic structure factor $S_{inc}(Q,\omega)$ is further convoluted with the instrumental resolution. The rotational and translational (or diffusive) terms are generally assumed to be independent, if the proton motion is decoupled, and described by Lorentzian functions, whose linewidths are respectively Q-independent or Q-dependent. For rotational or confined translational movements, the QENS signal is generally described as a $S(Q,\omega) = A_0(Q,T)\ \delta(\omega)+(1-A_0(Q,T)L(Q,T,\omega)$, where the first term accounts for the elastic, and the second for the quasi-elastic components. The elastic incoherent structure factor (EISF) $\equiv elastic_{intensity}/total_{intensity}$ associated with a relaxation characterises the geometry of the motion. Great care is however necessary to ensure the validity of all model assumptions, and ascertain the appropriate fractions of mobile and immobile protons, in particular in mixtures such as PA/H_2O membranes where the polymer network can be expected to undergo local (rotational) relaxations and water can potentially exhibit both bound and unbound populations, and further undergo rotational and diffusive motion. We have therefore taken a step-by-step approach analysing first the dry PA membrane, followed by the D_2O hydrated PA sample, where only polymer motion is measured experimentally, before tackling H_2O hydrated PA sample, where the polymer contribution was fixed based on the D_2O-hydrated PA measurement. The next sections detail how the complete model was constructed.

Supplementary Note 4. *Model for the dry polymer*

QENS analysis of the dry PA network (Supplementary Fig. 4-5) assumes that only vibrational and rotational relaxation motions are present, which is confirmed experimentally by the Q-independence of the Lorentzian linewidth Γ of the QENS signal. The geometry of the motion, and thus the associated EISF profile, was constructed based on the chemical structure of the PA units, shown in Fig. 2c of the main paper. Only a fraction of the polymer protons are mobile (ϕ_P^m), as illustrated in the main paper, as backbone protons are effectively immobile in the timescales probed.

Best agreement with data was found with for a modelled considering an out-of-plane partial rotation, or a 'flip', between two positions, with populations p_1 and p_2 (Fig. 2d-e, main paper), following earlier work [4].

Our system counts twelve hydrogen atoms per PA unit; of these only the protons directly connected to the MPD ring (which are six) can undergo an out-of-plane flip. Of these six, however, only five are more than 3 Å apart the plane of the polymer unity (highlighted in green in Fig. 2c, main paper), and we therefore approximate our system as composed by five mobile over a total of twelve protons, writing:

$$EISF_P = A_{0,P} = \frac{7}{12} + \frac{5}{12}[p_1 p_1 + p_2 p_2 + 2 p_1 p_2 j_0(Qd)] \tag{S6}$$

where j_0 is the Bessel function of the zeroth order, d is the distance between two positions and p_1 and p_2 the different occupation probabilities (where p_2 = 1-p_1).

Supplementary Fig. 6 reports a number of models considered for the apparent EISF for the dry PA, including: (i) free rotation in a sphere of radius r ($A_0 = j_0^2$ (Qr); Supplementary Fig. 6c,f; for BS and TOF data, respectively); (ii) jump among two equivalent sites ($A_0 = \frac{1}{2}$ ($1 + j_0^2$ (Qd)); where d is the jump distance; Supplementary Fig. 6b,e; for BS and TOF data, respectively); (iii) out-of-plane, two state, flip obtained by varying either the relative population of the two states or the angle of flipping (Supplementary Fig. 6d,g; for BS and TOF data, respectively).

Using two QENS energy resolutions, we find that the flipping of the amide ring is characterised by two different amplitudes when observed over the two different time scales. Specifically, the amplitude increases from $\simeq 15°$ (small amplitude; SA) to $\simeq 40°$ (larger amplitude motion; LA) when observed at the ps or ns timescale, respectively, as well as a different fraction of mobile protons involved in the relaxation (Fig. 3, main paper). To corroborate these findings, molecular dynamics simulations [5] were performed on PA membranes with TMC/MPD 1:1.5 stoichiometry, created *in silico* by interfacial polymerization (IP). The simulation confirms a spectrum of partial rotations compatible with the two different amplitudes and timescales found in experiment, as shown in Supplementary Fig. 6h-j.

As described above, the polymer is modelled as undergoing two types of motion: a small amplitude (SA), fast relaxation motion, well captured by TOF; and a slower, larger amplitude motion (LA), well resolved by BS. We expect that a fraction of protons $(1 - f)$ undergo SA motion only, while another fraction f undergo both SA and LA motion, which is corroborated by MD simulation. We therefore write the polymer term as:

$$S_P(Q,\omega) = (1 - \phi_P^m)\delta(\omega) + \phi_P^m \left[(1-f) S_{P_{SA}}(Q,\omega) + f\left(S_{P_{SA}}(Q,\omega) + S_{P_{LA}}(Q,\omega)\right)\right] \tag{S7}$$

which simplifies into

$$S_P(Q,\omega) = (1 - \phi_P^m)\delta(\omega) + \phi_P^m S_{P_{SA}}(Q,\omega) + \phi_P^m f S_{P_{LA}}(Q,\omega) \tag{S8}$$

and thus the fraction of SA-active protons is thus ϕ_P^m while the fraction of LA-active protons is $\phi_P^m \times f$.

In backscattering (BS) timescales, all protons undergoing small amplitude motions are active and faster than those captured by the experimental window, and thus the SA term appears as a flat background $B(Q)$,

$$S_P^{BS}(Q,\omega) = (1 - \phi_P^m)\delta(\omega) + \phi_P^m \left[B(Q) + f S_{P_{LA}}(Q,\omega)\right] \tag{S9}$$

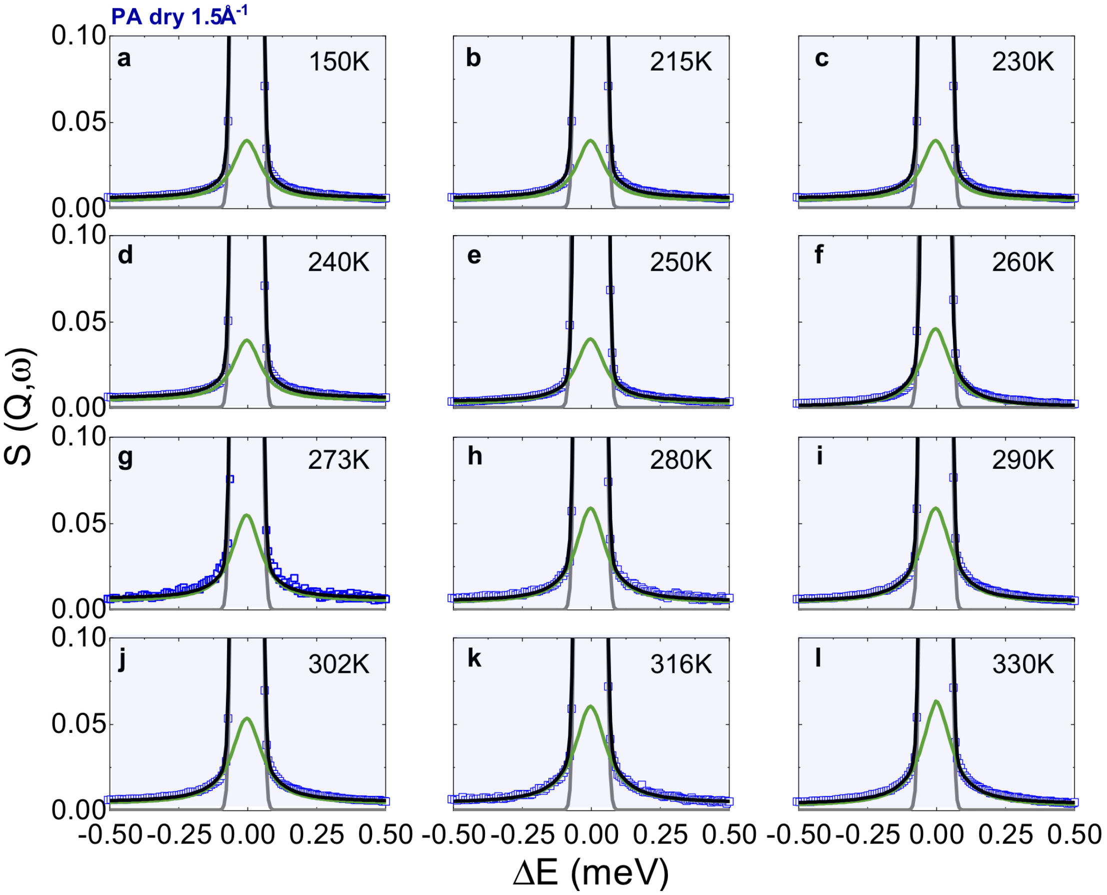

Supplementary Figure 4: Time-of-flight (IN5) data at $Q = 1.5$ Å^{-1} for PA in its dry state as a function of temperature, from 150 to 330 K. In each panel is reported the S(Q,ω) with the relative fit. In black is reported the global fit; in grey the instrumental resolution; in green the Lorentzian function describing the polymer segmental relaxation. The blue shading indicates that only polymer relaxations are visible. Source data are available at DOI:10.5291/ILL-DATA.9-11-1809 and 10.5291/ILL-DATA.9-11-5311718.

By contrast, in TOF timescales, a vanishingly small fraction of protons undergo large amplitude motions, and thus the LA term appears effectively as a $\delta(\omega)$ function, accounting for this apparently 'frozen' population:

$$S_P^{TOF}(Q,\omega) = (1-\phi_P^m)\delta(\omega) + \phi_P^m\left[S_{P_{SA}}(Q,\omega) + f\delta(\omega)_{LA}\right] = [(1-\phi_P^m) + \phi_P^m f]\delta(\omega) + \phi_P^m S_{P_{SA}}(Q,\omega) \quad \text{(S10)}$$

Experimentally (330K), we find by TOF an (apparent) mobile fraction of 0.19 (dry), and from BS, we find a mobile fraction of 0.95 (Supplementary Fig. 8); thus $\phi_P^m(1-f) = 0.19$ and $\phi_P^m(B+f) = 0.95$ (but (B+f) = 1 anyway), and therefore, $f \simeq 0.8$. Explicitly considering a background term $B(Q)$, effectively appearing inside the bracket in Eq. (S9) above, the actually fitted expression reads:

$$S_P^{BS}(Q,\omega) = (1-\phi_P^m)\delta(\omega) + \phi_P^m\left[f S_{P_{LA}}(Q,\omega)\right] + B(Q) \quad \text{(S11)}$$

In this case, the *apparent* mobile fraction in BS would be 0.95; in the limit that $\phi_P^m(B+f)$=1, we find f=0.81 so this difference is inconsequential.

Supplementary Note 5. *Model for the hydrated polymer in D_2O*

To examine the polymer in hydrated state, a sample hydrated in D_2O saturated vapor environment was measured at the two resolutions and a series of temperatures. The negligible incoherent contribution of heavy water contribution enables the polymer signal to be isolated, yet permits the study of the polymer dynamics in its swollen state. The QENS data, shown in Fig. 4a,c,e of the main paper and Supplementary Fig. 5,7,8 (profiles in purple) indicate that the dynamics can be described as a rotational relaxation, as in the case of the dry polymer albeit with different mobile population and frequency. From the EISF data fitting, we conclude that the geometry of the rotational relaxation of polymer in dry and D_2O-hydrated state, remains unchanged within measurement uncertainty (Fig. 3c,d and 4e, main paper, and Supplementary Fig. 8-9). Applying similar approach as in the case of the dry polymer, for PA-D_2O we find that only ϕ_P^m changes with temperature (0.71, 0.81, 0.89 and 0.95 at 230, 260, 290 and 330 K) and f does not ($\simeq$ 0.8).

Our experiments indicate an activation energy (E_a) of 1.2 kJ mol^{-1} (Supplementary Fig. 9g). This value is approximately one order of magnitude lower than those reported for γ-relaxation for instance in nylon or polystyrene [6, 7, 8] which, however, generally exhibit a broad activation energy distribution associated with the various local conformations of the backbone chain that affect ring flip motions. Upon D_2O-hydration, E_a decreases slightly to 1.1 kJ mol^{-1} (Supplementary Fig. 9g) compatible with the expectation of water uptake and the membrane swelling.

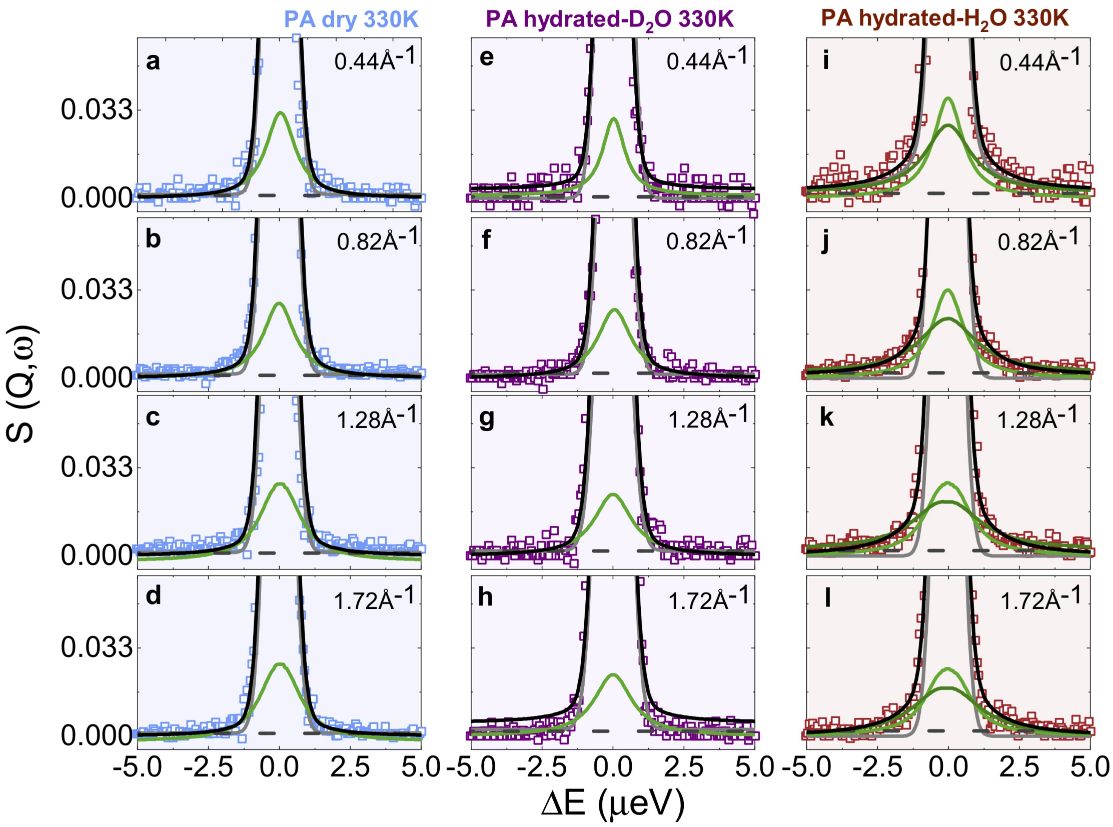

Supplementary Figure 5: Backscattering (IN16B) data (at $Q = 0.44$, 0.82, 1.28, and 1.7 Å^{-1}) for PA in its dry (left panel) and hydrated state (in H_2O and D_2O; middle and right panel, respectively). In each panel is reported the S(Q,ω) with the relative fit. In black is reported the global fit; in grey the instrumental resolution (1 μeV); in green the Lorentzian function (light and dark green for 1st and 2nd, respectively).The blue and purple shading indicate that only polymer relaxations are visible, while the red shading indicates that diffusive water motion is also visible. Source data are available at DOI:10.5291/ILL-DATA.9-11-1809 and 10.5291/ILL-DATA.9-11-5311718.

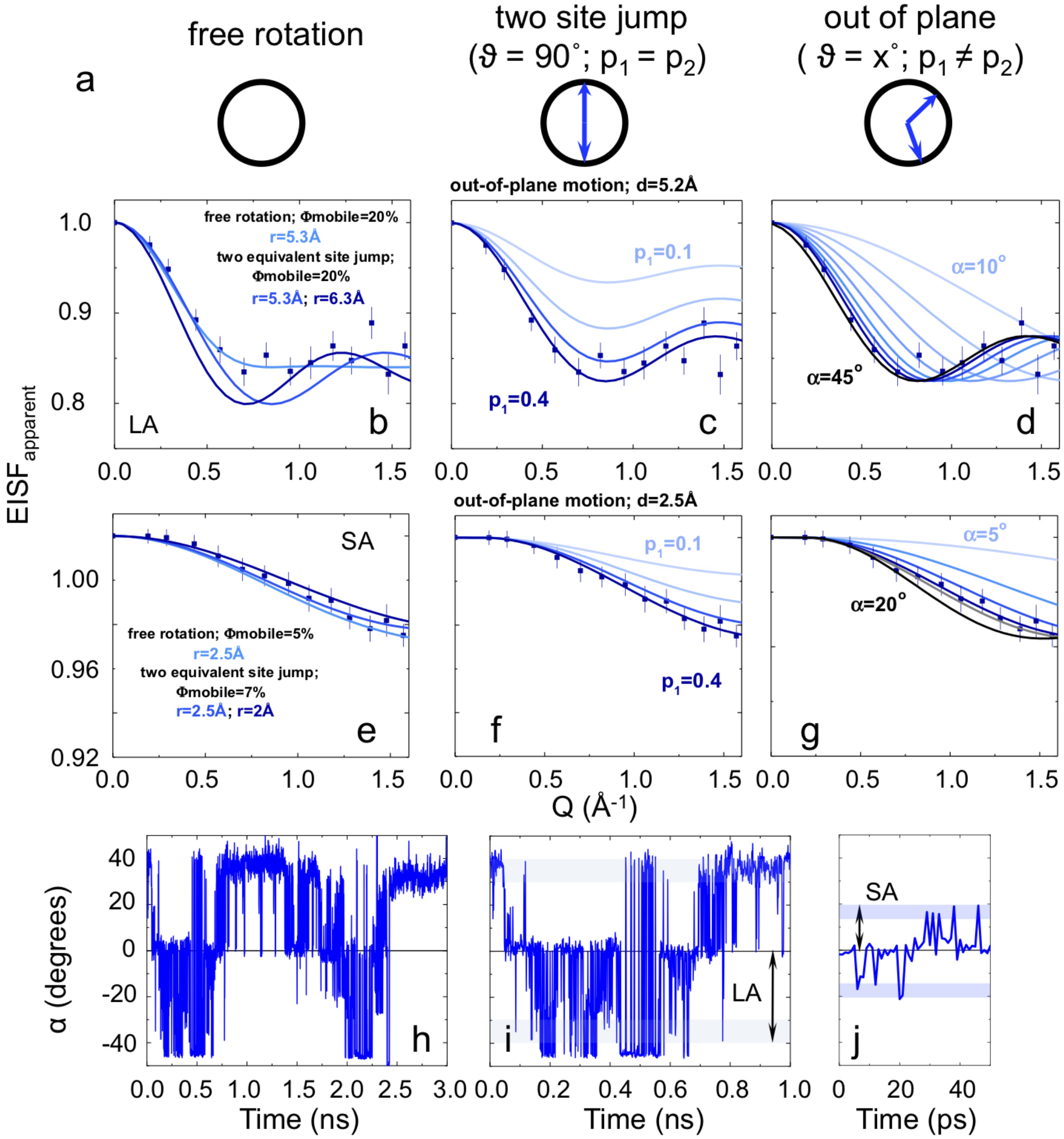

Supplementary Figure 6: Apparent EISF for free rotation, jump between two equivalent sites and out-of-plane, two-state, flip of the MPD ring in the PA dry polymer, computed by eqs. S6-S11 above. a) Cartoon representing the various models; b, e) the apparent EISF modelled by considering either a free rotation or jump diffusion among two equivalent sites (panels b and e; for BS and TOF data, respectively) [3]; c, f) the apparent EISF modelled by considering a fixed angle ($\alpha = 40°$ and $14°$, for BS and TOF data, respectively) of out-of-plane flip and varying the relative population of the two states (p_1 and p_2) [3]; d, g) apparent EISF modelled by considering a fixed relative population of the two states (40% - 60%, for both BS and TOF data) of out-of-plane flip and varying the flipping angle ($10° < \alpha < 45°$ and $5° < \alpha < 20°$, for BS and TOF data, respectively) [4]. h-j) Time variation of the phenyl group angle of flip (α; see Fig. 2d, main paper) for cross-linked PA membrane in its dry state obtained from MD [5]. h) Simulation within 3 ns time frame, while (i) depicts a 1 ns time frame which is equivalent to the time scale investigated by BS ($\alpha \simeq 40°$; Large Amplitude *LA*), and (j) shows a 50 ps time frame, commensurate with the time scales investigated by TOF ($\alpha \simeq 15°$; Small Amplitude *SA*). These results are compatible with the model (out-of-plane, two-state, flip) employed to model the EISF of the PA dry polymer. Source data file provided.

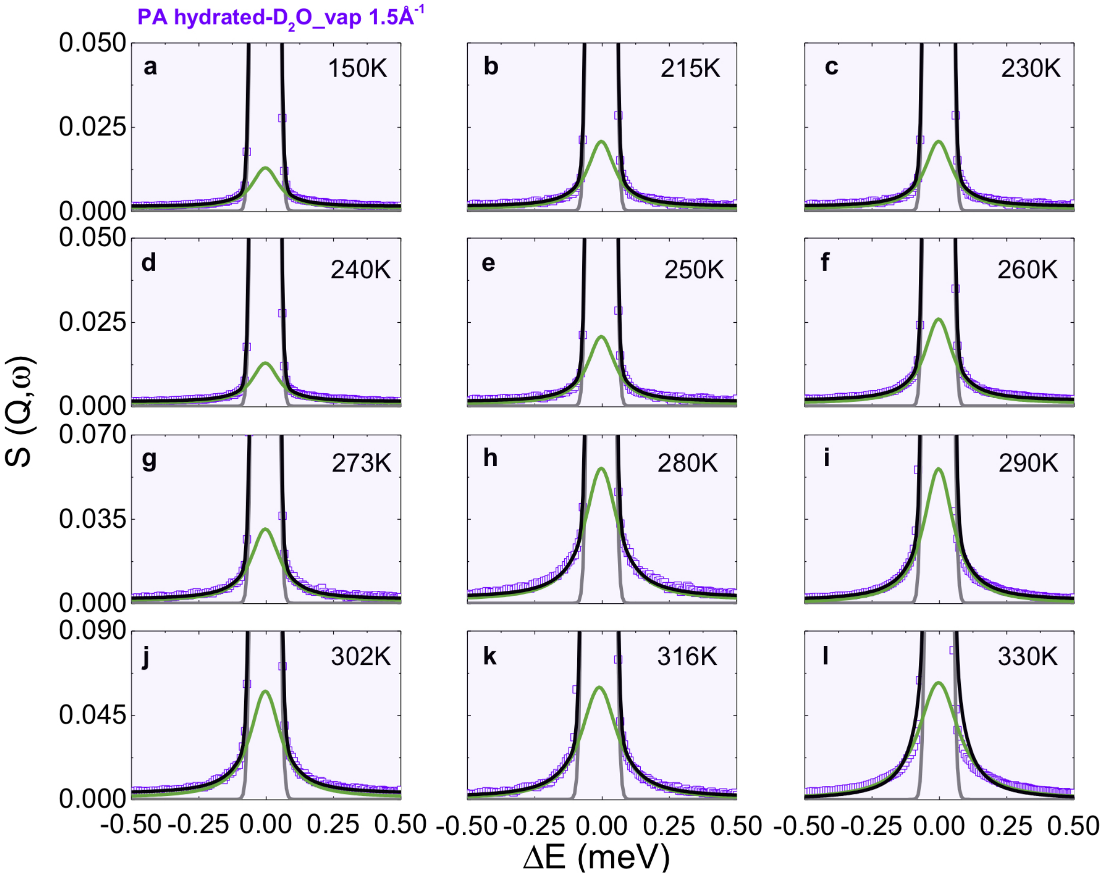

Supplementary Figure 7: Time-of-flight (IN5) data at $Q = 1.5$ Å^{-1} for PA hydrated in D_2O as a function of temperature, from 150 to 330 K. In each panel is reported the S(Q,ω) with the relative fit. The black lines indicate the global fit; in grey the instrumental resolution (45 μeV); in green the Lorentzian function describing the polymer segmental relaxation. The purple shading indicates that only PA relaxation is visible. Source data are available at DOI:10.5291/ILL-DATA.9-11-1809 and 10.5291/ILL-DATA.9-11-5311718.

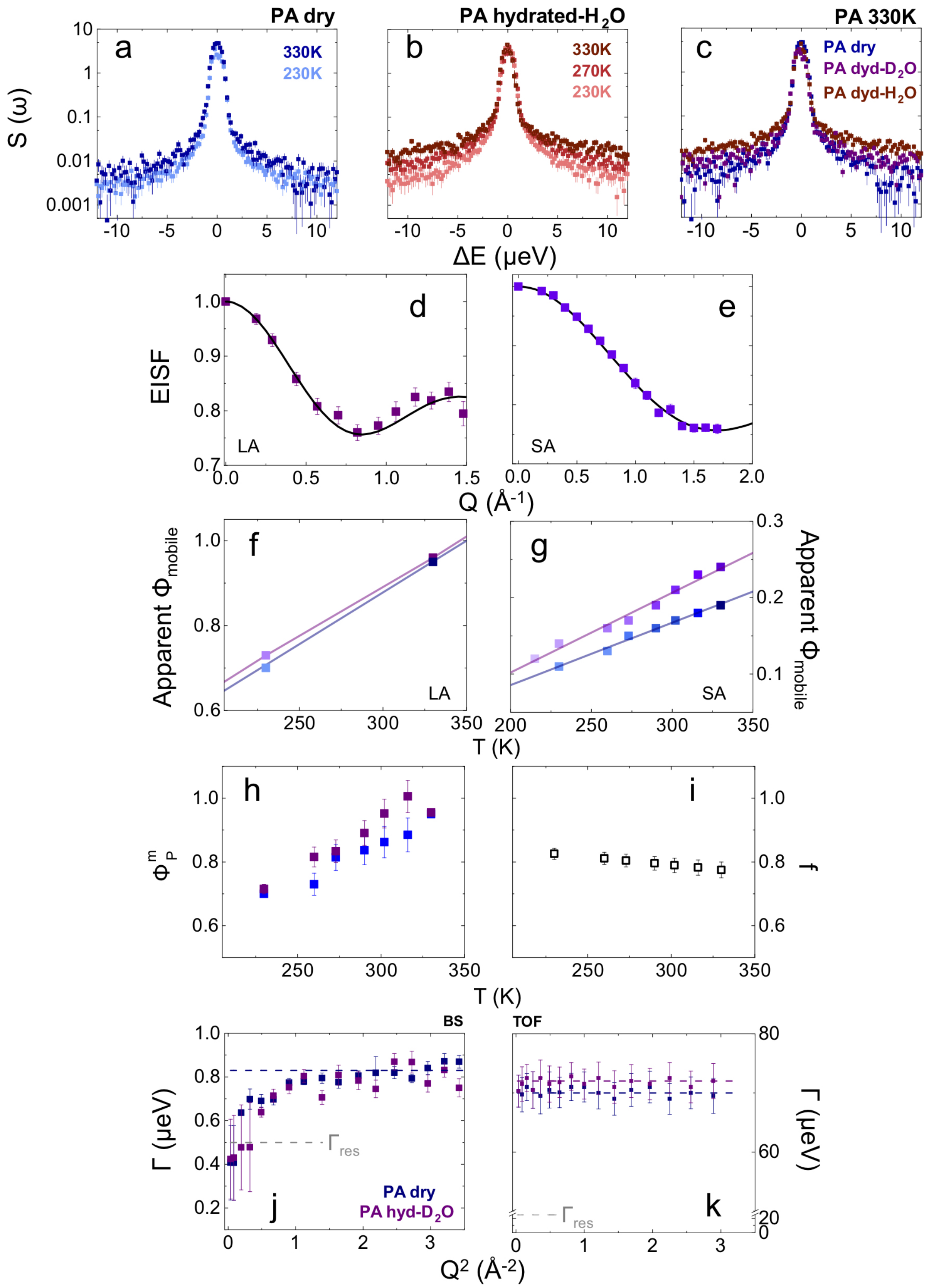

Supplementary Figure 8: (a-c) BS data, integrated over the entire Q-range, for PA in its dry (blue), hydrated in D_2O (purple) and H_2O (red). a) Comparison between PA dry samples at 230 and 300 K. b) Comparison between samples hydrated in H_2O at 230, 270 and 300 K. c) Comparison between dry and hydrated samples at 300 K. d-e) EISF (accounting for the percentage of mobile protons in the system) for the out-of-plane flip of MPD rings in PA hydrated with D_2O. d) EISF data and fit obtained at 1 μeV resolution (BS); e) EISF data and fit obtained at 45 μeV resolution (TOF). f-g) Show the fraction of mobile protons, obtained in BS and TOF windows; the solid line (without data points) corresponds to the dry membrane. h,i) Proton fraction undergo SA motion only $(1-f)$ or both SA and LA motion (f). j) Half width at half maximum (HWHM) $\Gamma(Q^2)$ for PA in its dry (blue) and D_2O-hydrated (purple) at 330 K, recorded at 1 μeV resolution (BS) and k) at 45 μeV resolution (TOF). The corresponding instrumental resolutions are shown by the horizontal dashed lines (Γ_{res} = 0.5 and 22.5 μeV). Source data are available at DOI:10.5291/ILL-DATA.9-11-1809 and 10.5291/ILL-DATA.9-11-5311718, and provided as a Source Data file.

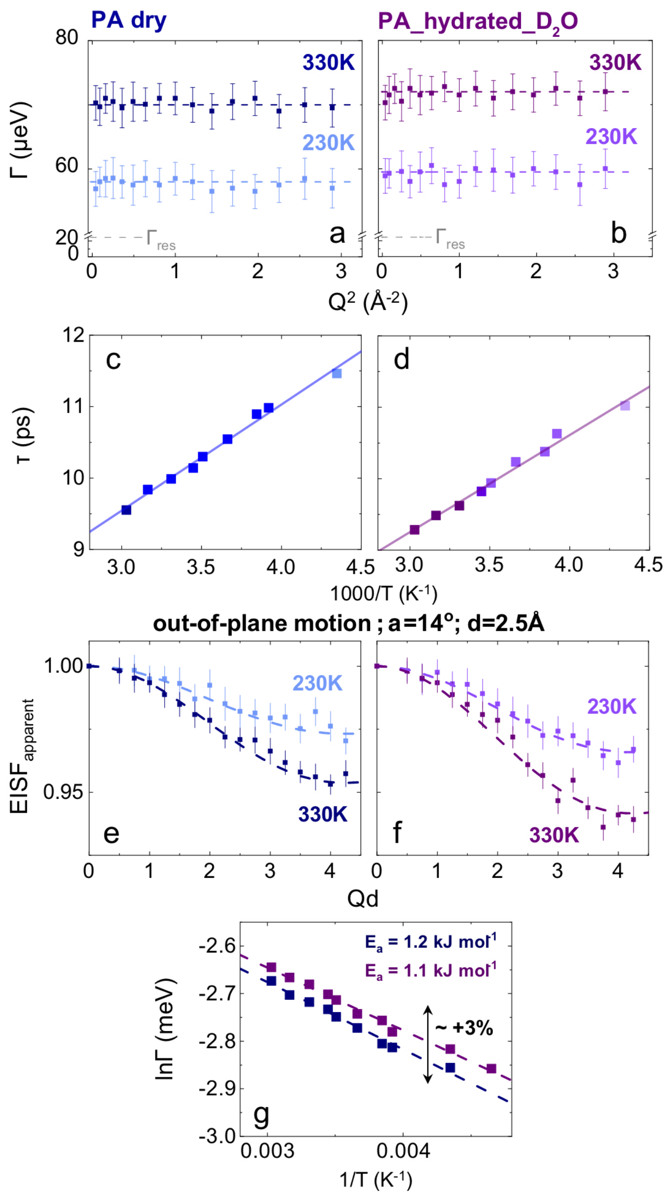

Supplementary Figure 9: a-b) Half width at half maximum (HWHM) $\Gamma(Q^2)$ obtained for both the (a) PA dry polymer and (b) fully hydrated in D_2O vapor at selected temperatures 230 and 330 K, the instrumental (half) resolution Γ = 22.5μeV is shown in grey. c-d). The corresponding rotational relaxation times computed by $\tau_0 = \hbar/\Gamma_P^R$. e-f) EISF apparent for an out-of-plane, two-state, flip and model fit [4] of the MPD ring in the PA dry polymer (model 1) hydrated in D_2O vapor (model 2). g) Arrhenius behaviour for $\Gamma = \Gamma_0 \exp(-E_a/KT)$) and associated activation energy (E_a) associated with the polymer segmental relaxation, for both the dry PA polymer (blue) and fully hydrated in D_2O vapor (purple). Source data file provided.

Supplementary Note 6. *Model for the hydrated polymer in H_2O*

We have further extended the study to the H_2O-hydrated PA membrane; as before, we have employed two energy resolutions and tested the sample over a series of temperatures. Below 230 K the dynamics can be described as a rotational relaxation (as in the case of both dry and D_2O-hydrated PA membranes; Supplementary Fig. 10-11). H_2O dynamics become visible above 230 K (Supplementary Fig. 12-13).

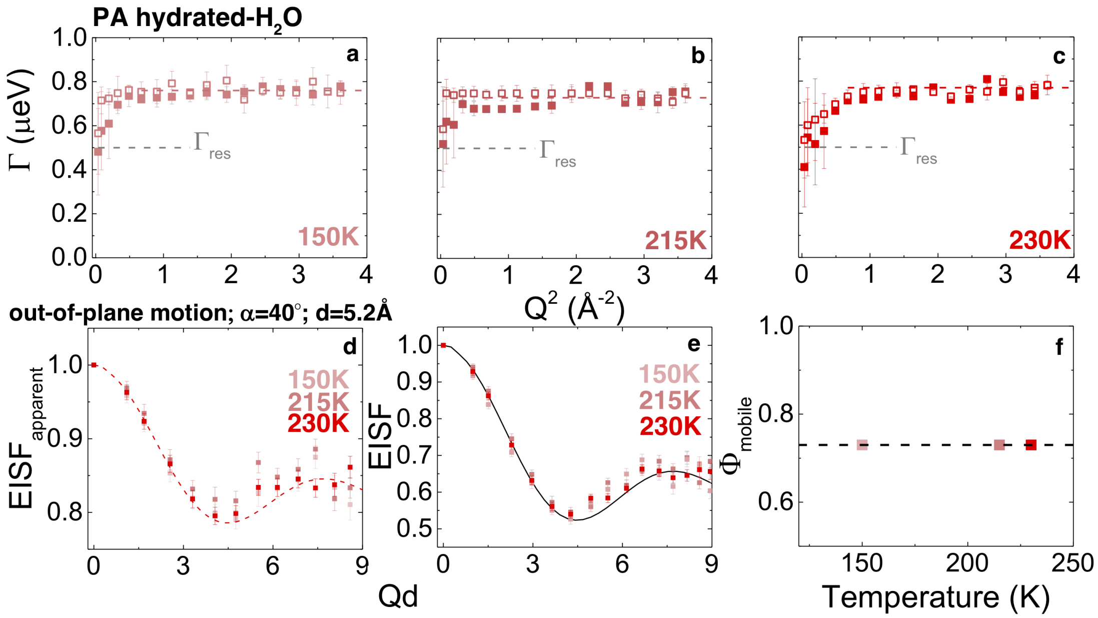

Supplementary Figure 10: a-c) Half width at half maximum $\Gamma(Q^2)$ for H_2O vapor hydrated PA at three selected temperatures, 150, 215 and 230 K; the grey line shows the instrumental (half) resolution ($\Gamma = 0.5\mu$eV). d) EISF apparent for an out-of-plane, two-state, flip of the MPD ring in the PA hydrated (in H_2O) polymer, under conditions where no contribution from water is visible. e) Corresponding EISF accounting for the fraction of mobile protons, shown in f Note that at these temperatures only the polymer contribution was detectable. Source data are provided as a Source Data file.

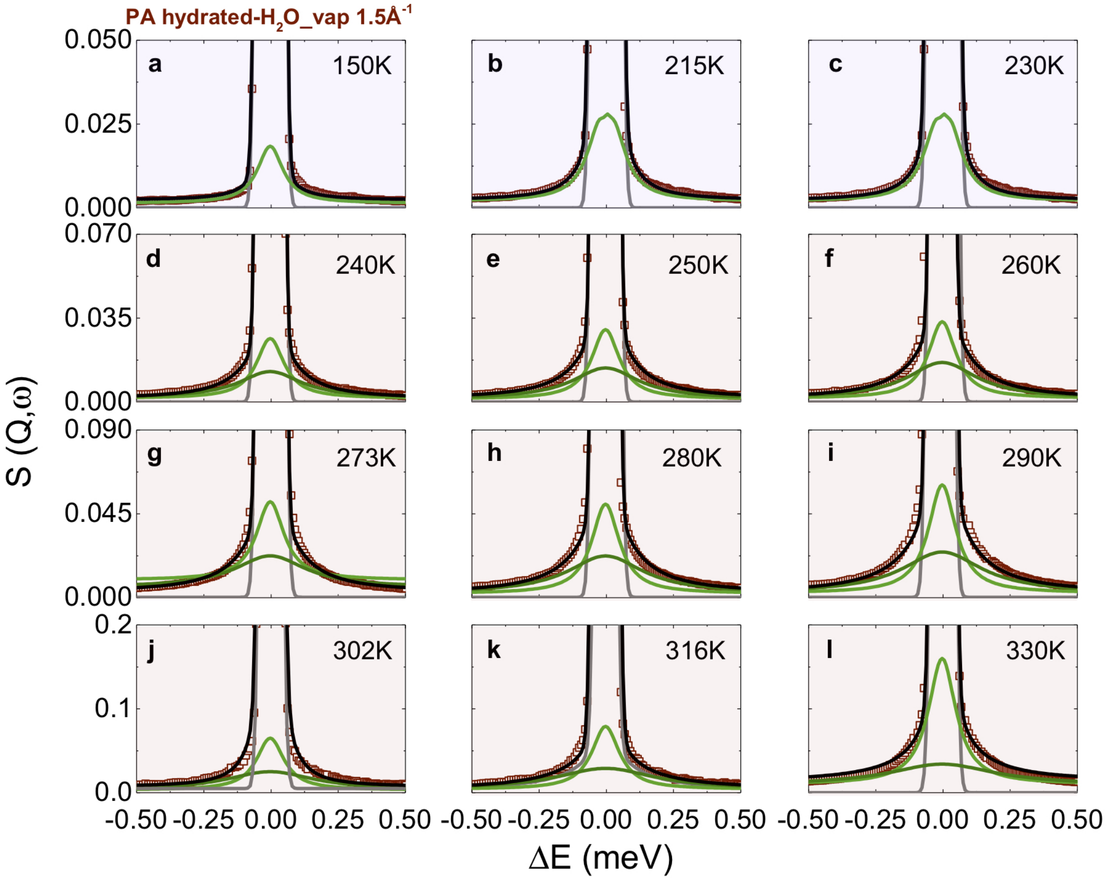

Supplementary Figure 11: Time of flight (IN5) data at Q $= 1.5$ Å^{-1} for H_2O hydrated PA as a function of temperature, from 150 to 330 K. In each panel is reported the S(Q,ω) with the relative fit. In black is reported the global fit; in grey the instrumental resolution (45 μeV); in light green the Lorentzian function (1st, narrower) describing the polymer segmental relaxation and in dark green the Lorentzian function (2nd, broader) describing the water dynamics. The purple shading indicates that only PA segmental relaxation is visible, while red indicates that water diffusion is also present. Source data are available at DOI:10.5291/ILL-DATA.9-11-1809 and 10.5291/ILL-DATA.9-11-5311718.

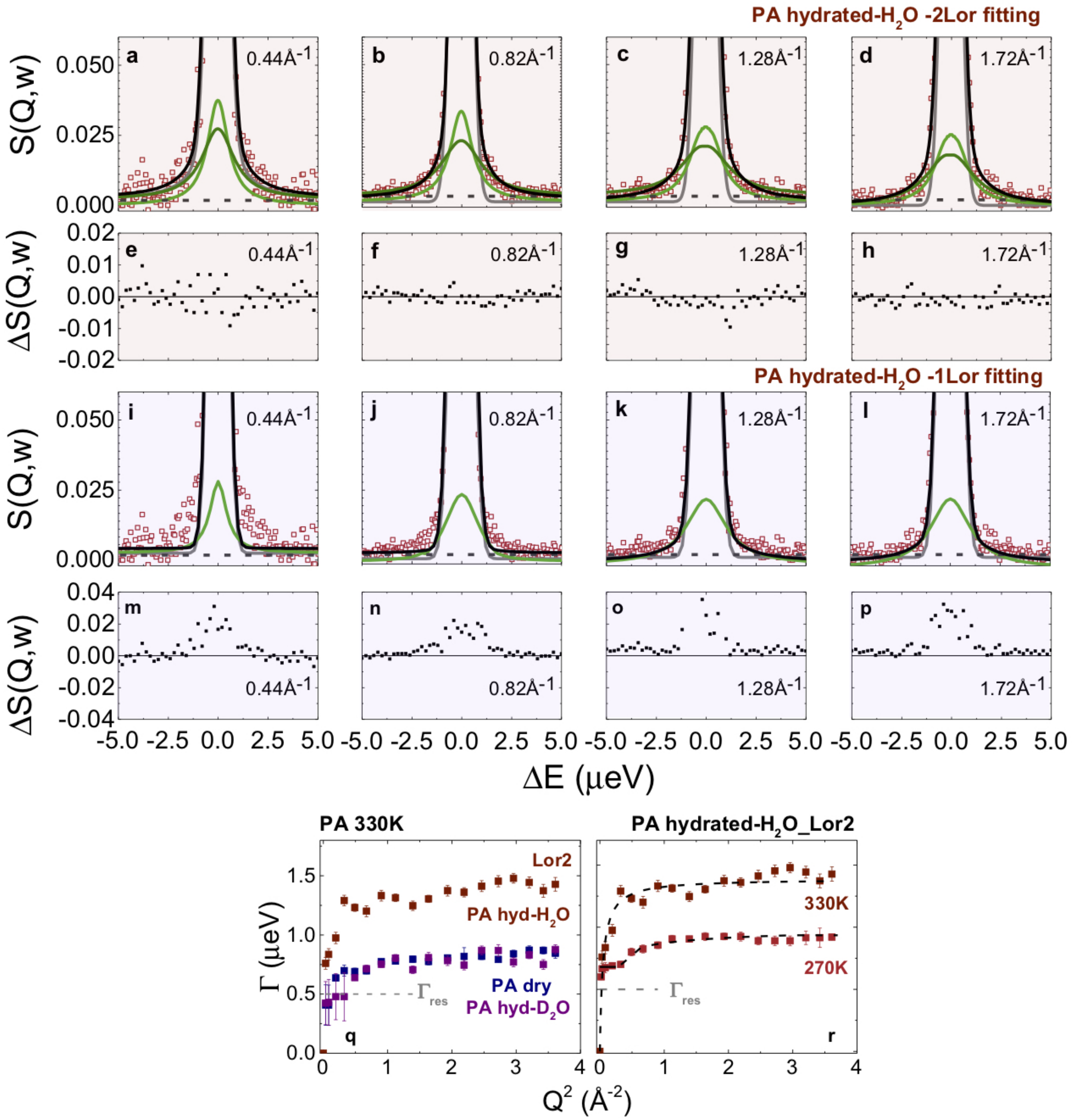

Supplementary Figure 12: a-p) BS data measured at Q = 0.44, 0.82, 1.28, and 1.72 $Å^{-1}$ for H_2O vapor hydrated PA at 330 K. Panels a-d show S(Q,ω) with the relative fit, considering two Lorentzian functions, while panels (e-h) show the difference between data and model fit. Panels i-l show S(Q,ω) with the relative fit, considering now only one Lorentzian function, accompanied by the difference between model and data in panels (m-p). The purple shading indicates only PA relaxation (and thus only one Lorentzian function to model the data) while the red shading indicates also water diffusion, and thus two Lorentzians functions to model the data). q) Comparison between $\Gamma(Q^2)$ corresponding to the polymer relaxation at 330K in its dry (blue) and D_2O hydrated (purple) state, alongside the contribution from H_2O signal (red), in this case obtained from the second Lorentzian. r) $\Gamma(Q^2)$ from the 2nd Lorentzian function fitted (black dashed line; Eq. 8 main paper) to H_2O hydrated PA samples at two selected temperatures. The grey dashed line shows the instrumental (half) resolution Γ_{res}= 0.5 μeV. Source data are available at DOI:10.5291/ILL-DATA.9-11-1809 and 10.5291/ILL-DATA.9-11-5311718. Source data for (q,r) are provided as a Source Data file.

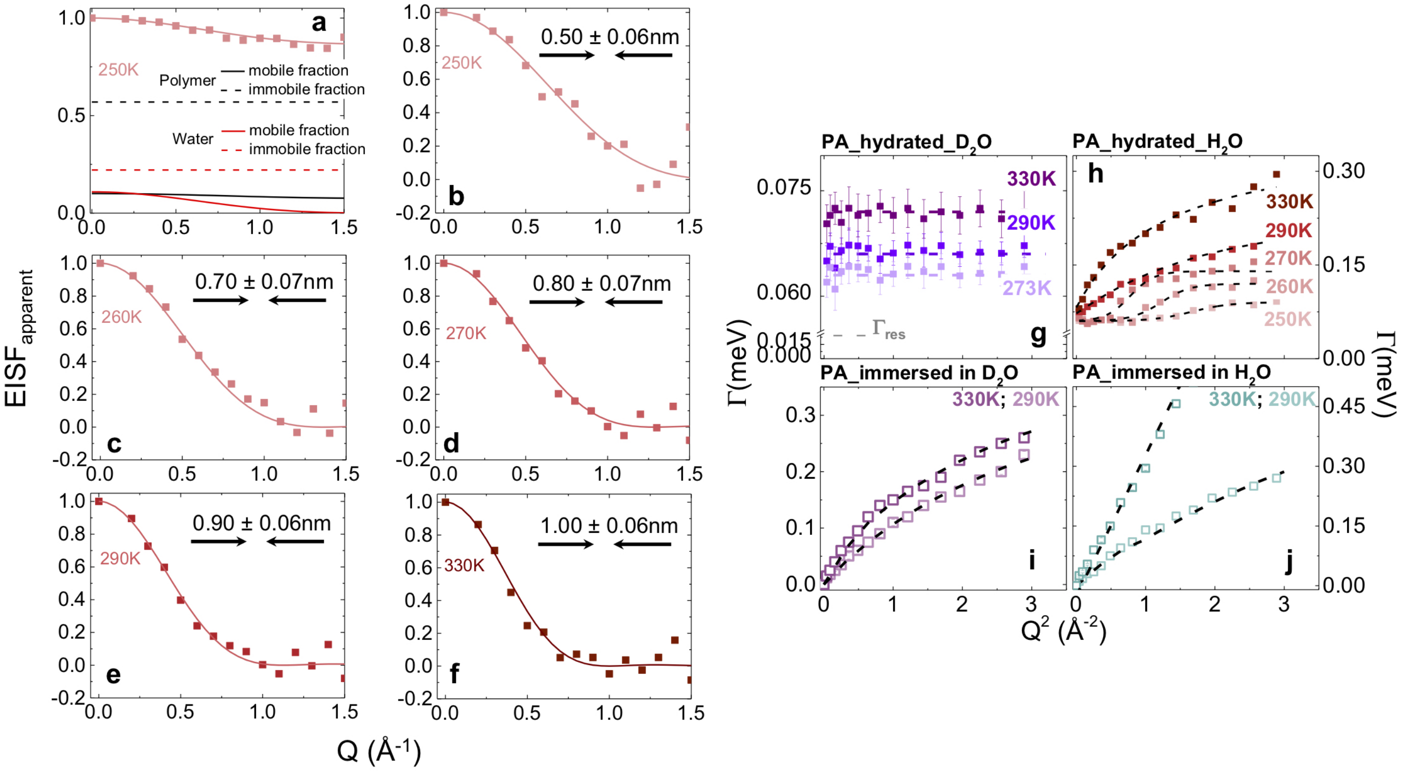

Supplementary Figure 13: Time-of-flight analysis of hydrated PA samples, in both H_2O and D_2O vapour and liquid (immersed). Specifically, panels (a-f) correspond to H_2O vapour; (g) D_2O vapour; (h) H_2O vapour; (i) immersed in D_2O; and (j) immersed in H_2O. a) Apparent EISF describing the out-of-plane, two-state, flip of the MPD ring and diffusion of water molecule inside of a sphere or radius a (model 3) obtained for the H_2O hydrated polymer sample at 250K. The polymer EISF component was fixed from D_2O hydrated PA measurement. Panels (b-f) show only the measured EISF corresponding to the water diffusion in confinement, at selected temperatures 250, 260, 270, 290 and 330 K showing the variation in confinement diameter $2a$. g-j) Time of flight data comparing $\Gamma(Q^2)$ for the PA polymer hydrated in (g) D_2O and (h) H_2O, as well as liquid immersed PA samples in (i) D_2O and (j) H_2O highlighting the importance of the hydration process. The grey dashed line shows the instrumental (half) resolution Γ_{res}= 22.5 μeV. In the case of the hydrated in D_2O-vapour sample, only one Lorentzian function was required to fit $S(Q,\omega)$ shown in (g) while two Lorentzians were employed in the remaining cases (h-j) whose Γ is plotted. Given the excess of water in direct liquid immersion PA samples, a weak signal from D_2O diffusion can be detected and its Γ is shown in panel (i); incidentally this diffusion profile differs from that of H_2O in analogous conditions due to the difference of density and bond length in heavy water [9]. Source data are provided as a Source Data file.

Upon H_2O hydration of the PA membrane, contributions from the polymer membrane, as well as the water dynamics must be considered in the dynamic structure factor. The experimental elucidation of the resulting, complex $S(Q,\omega)$ requires the combination of experiments involving selective deuteration (PA-D_2O and PA-H_2O) and two different spectrometer resolutions. In simple terms, 4 equations (2 contrasts $\times$ 2 resolutions) are needed to resolve the complex $S(Q,\omega)$ of PA-H_2O hydrated membranes. The polymer rotational relaxations (small and large amplitude, SA and LA) are resolved experimentally by TOF and BS experiments of PA-D_2O specimens. Water dynamics are expected to include translational diffusion dynamics, potentially involving several populations of water molecules and/or mechanisms (rotational dynamics were found not to be necessary to account for the experimental data). Such models can become complex and care is needed to ensure self-consistency, as model solutions may not necessarily be single-valued.

In first approximation, S_{total}(Q,ω) is taken as a sum of two decoupled contributions: one describing the polymer and the other describing the water dynamics. As such, the dynamic structure factor is written as:

$$S_{total}(Q,\omega) = \varphi_P S_P(Q,\omega) + \varphi_W S_W(Q,\omega) \tag{S12}$$

where φ_P and φ_W are the proton fractions of polymer and water ($\varphi_P + \varphi_W$=1), and $S_P(Q,\omega)$ and $S_W(Q,\omega)$ are the corresponding polymer and water dynamic structure factors.

Polymer dynamics, as described above, is modelled as undergoing two types of motion: a small amplitude (SA), fast relaxation motion, well captured by TOF; and a slower, larger amplitude motion (LA), well resolved by BS.

Water dynamics are tentatively described in terms of a series of translational diffusion processes which we associate to the heterogeneous nature of the membrane, and imposed confinement at the nanoscale. Assuming the processes to be decoupled, the dynamic structure factor is written with as a sum of terms,

$$S_W(Q,\omega) = (1-\phi_W^m)\delta(\omega) + \phi_W^m \left[(1-g)S_W^{tr}(Q,\omega) + g\left(S_W^{tr}(Q,\omega) + S_W^{lr}(Q,\omega)\right)\right] \tag{S13}$$

where the first term accounts for the immobile water fraction (bound water), the second term accounts for water molecules that undergo solely translational (tr) diffusion, and the the final term arises from molecules which undergo *both* translational (tr) and long-range (lr) diffusion. This expression simplifies into eq. (6) of the main paper. In TOF experiments, term $S_W^{tr}(Q,\omega)$ dominates the measured signal, and $S_W^{lr}(Q,\omega)$ contribution appears within the elastic line. Conversely, in BS measurements, probing longer timescales, the signal is now dominated by $S_W^{lr}(Q,\omega)$ while the Lorenztian profile corresponding to $S_W^{tr}(Q,\omega)$ appears as a nearly flat background term $B(Q)$. The combination of BS and TOF thus provides a convenient way of decoupling the two contributions.

Amongst the mobile water population, a small fraction g of molecules is modelled to undergo both tr and lr diffusion, while the majority of water molecules $1-g$ undergoes only tr diffusion in the time scales probed. The translational diffusion of water is well described by a jump-diffusion model [10, 3]

$$S_W^{tr}(Q,\omega) = \frac{1}{\pi}\frac{\Gamma_W^{tr}}{\omega^2 + (\Gamma_W^{tr})^2} \tag{S14}$$

where the linewidth (half width at half maximum) is

$$\Gamma_W^{tr} = \frac{D_{tr}Q^2}{1 + D_{tr}Q^2\tau_0^{tr}} \tag{S15}$$

and D_{tr} is the familiar translational diffusion coefficient or water D_{tr} (in the limit of large distances, Q→0, or high temperature, Fickian diffusion $\Gamma_W^{tr} = D_{tr}Q^2$ is recovered). Parameter τ_0 is the residence time between jumps, and the mean mean jump length is

$$l^{tr} = \sqrt{6D_{tr}\tau_0^{tr}} \tag{S16}$$

At lower temperatures, evidence of spatial confinement emerges in the form of an additional elastic component $\delta(\omega)$ and linewidth Γ that becomes invariant at low-Q, and the dynamic structure factor is generalised to

$$S_W^{tr}(Q,\omega) = A_{0,W}^0(Q)\delta(\omega) + (1 - A_{0,W}(Q))\frac{1}{\pi}\frac{\Gamma_W^{tr}}{\omega^2 + (\Gamma_W^{tr})^2} \tag{S17}$$

where, for the diffusion of a point particle inside a sphere of radius a, the EISF reads [11]

$$EISF_W \equiv A_{0,W}^0(Qa) = \left(\frac{3j_1(Qa)}{Qa}\right)^2 \tag{S18}$$

where j_1 is a spherical Bessel function. At low Q ($< \pi/a$), the linewidth exhibits a plateau value

$$\Gamma_W^{tr}(Q \to 0) \equiv \Gamma_0 = 4.33\frac{D_{loc}}{a^2} \tag{S19}$$

defining a local diffusion coefficient, D_{loc}, within the confinement region, and an EISF characteristic of the geometry of the confining volume. The confining radius (and effective 'pore diameter' estimated by $d \simeq 2a$) can thus be obtained, separately, from the EISF as well as from the Q threshold below which the linewidth Γ_W^{tr} becomes constant.

The long-range translational diffusion motion can be modelled in a similar fashion:

$$S_W^{lr}(Q,\omega) = \frac{1}{\pi}\frac{\Gamma_W^{lr}}{\omega^2 + (\Gamma_W^{lr})^2} \tag{S20}$$

with linewidth (HWHM):

$$\Gamma_W^{lr} = \frac{D_{lr}Q^2}{1 + D_{lr}Q^2\tau_0^{lr}} \tag{S21}$$

and lr diffusion coefficient, D_{lr}, and jump length $l^{lr} = \sqrt{6D_{lr}\tau_0^{lr}}$.

Experimentally, we find that tr and lr motions appear to be decoupled and can be well resolved by combining TOF and BS experiments. TOF data for the water contribution were found to be well described by a single Lorentzian profile with

$$S_W^{TOF}(Q,\omega) = (1 - \phi_W^m)\delta(\omega) + \phi_W^m\left[(1-g)S_W^{tr}(Q,\omega) + g\delta_W^{lr}(\omega)\right] \tag{S22}$$

in addition to the polymer contribution discussed above, which previously resolved from the TOF analysis of PA-D_2O specimens, in which water dynamics are not visible, and thus fixed in the analysis of PA-H_2O membrane dynamics. At the higher measured temperatures, we find that the mobile fraction $\phi_W^m(1-g) \simeq$ 0.8-0.9, and the remaining $(1-\phi_W^m) + \phi_W^m g$=0.2-0.1 is immobile. The 'apparent' EISF, measured experimentally at low temperature with a PA-H_2O sample, compounds contributions from the polymer and water dynamics. Since the polymer relaxation EISF is known, the EISF corresponding to confined water diffusion can be calculated from:

$$EISF_{apparent}^{TOF} = \Phi_P\left[(1-\phi_P^m) + \phi_P^m EISF_P\right] + \Phi_W\left[(1-\phi_W^m) + \phi_W^m\left[(1-g)EISF_W + g\right]\right] \tag{S23}$$

The BS data analysis can similarly be simplified by noting that the tr contribution becomes very broad and thus appears as a flat background $B(Q)$, yielding

$$S_W^{BS}(Q,\omega) = (1-\phi_W^m)\delta(\omega) + \phi_W^m\left[B(Q) + gS_W^{lr}(Q,\omega)\right] \tag{S24}$$

We find that explicitly fixing $S_W^{tr}(Q,\omega)$ obtained from TOF, instead of a flat $B(Q)$ profile does not change the LR fitting parameters, and we thus opt for considering just a background. Experimentally, the 'apparent' EISF measured by BS for PA-H_2O samples also subsumes contributions from both the polymer and the immobile water fraction, and we thus write:

$$EISF_{apparent}^{BS} = \Phi_P\left[(1-\phi_P^m) + \phi_P^m EISF_P\right] + \Phi_W\left[(1-\phi_W^m)\right] \tag{S25}$$

which is simpler than for TOF, since there is no EISF associated with lr translations.

Experimentally, at 330 K, in BS we find that the immobile water fraction is vanishingly small, and from Eq. (S24) we have $\phi_W^m \simeq 1$. Taking $\phi_W^m(1-g)$=0.8-0.9 from TOF measurements, we obtain $g \sim$ 0.2-0.1. This implies that the vast majority of water molecules are mobile over the measured timescales and that a fraction $g \simeq$ 0.2 undergoes LR motion.

Although we have not measured BS data at all temperatures, we have computed the TOF prefactors $\phi_W^m(1-g)$, which requires an assumption regarding the temperature dependence of ϕ_W^m and g to fully resolve the unknowns. Assuming (1) a linear dependence of the mobile fraction in temperature (this would give $\phi_W^m \simeq$ 0.0017 T [K] + 0.45), we would have ϕ_W^m(T=260K)$\simeq$ 0.892 and ϕ_W^m(T=250K)$\simeq$ 0.875. If correct, these would lead to g(T=260K)=1-0.60/0.892$\simeq$0.33 and g(T=250K)=1-0.40/0.875$\simeq$0.54. At 230K the TOF mobile fraction is zero, and this would require an unfeasible g $\sim$1. It appears therefore more plausible that ϕ_W^m has the strong temperature dependence and g is rather more constant. Assuming instead (2), that g $\simeq$0.20, we find self-consistent values for the fraction of mobile protons, compatible with TOF and BS data across the temperature range.

Equivalent analysis of the hydrated polymer and water dynamics in the time-domain. In order to corroborate the self-consistency and decoupling hypothesis of our analysis, we have Fourier-transformed representative BS and TOF data for the H_2O vapor-hydrated PA membranes in the time domain, as shown in Supplementary Fig. 14. The same model of analysis reported above is found to satisfactorily describe the data, and we thus opt to pursue the BS and TOF analysis directly in $S(Q,\omega)$ space to minimise data manipulations associated with transformation in time-domain.

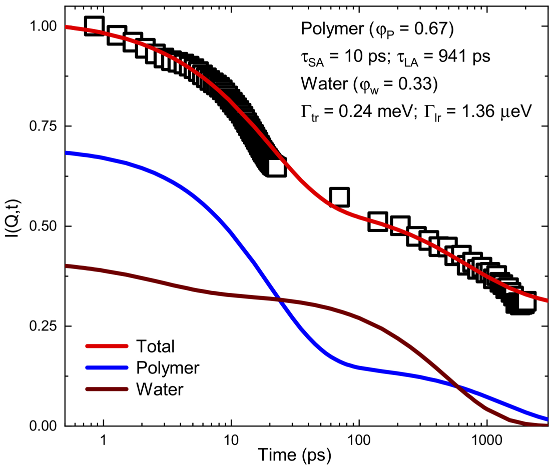

Supplementary Figure 14: Intermediate scattering function $I(Q,t)$ corresponding to the data for H_2O-vapor hydrated PA membranes at 330 K and Q = 1.5 Å^{-1} comparing the analysis in energy- and time-domain. Source data are available at DOI:10.5291/ILL-DATA.9-11-1809 and 10.5291/ILL-DATA.9-11-5311718.

Rotational-jump diffusion of water. We finally note that water dynamics are generally described by rotational jump diffusion models, in which translational and rotational diffusion processes are coupled, requiring two Lorenztian profiles to described TOF data for bulk water dynamics. In our measurements, we find that a single (translational) Lorentzian suffices to describe the data at the resolution employed, and that data fitting did not significantly improve with the added 'rotational' Lorentzian. We have therefore considered only translational dynamics in our analysis.

Possible correlation between polymer and water dynamics. Further, it is conceivable that polymer and water dynamics might be coupled, for instance if the relaxation of the polymer segment impacts the diffusion of water, since these occur (as found here) in commensurate timescales. The dynamic structure factor for such coupled systems is written instead as a convolution of terms in Fourier space,

$$S_{total}(Q,\omega) = \varphi_P S_P(Q,\omega) \otimes \varphi_W S_W(Q,\omega) \quad \text{(S26)}$$

However, since $S_P(Q,\omega)$ and $S_W(Q,\omega)$ comprise two terms each, it is not clear whether the dynamic coupling would take place between all or a few processes, and a number of combinations involving (partial) convolution and addition of terms could be envisaged. While our analysis cannot rule these out, herein we demonstrate that a self-consistent and compelling description of all experimental data (an unprecedented dataset of various contrasts, resolution, hydration, and temperature) can be described with the additive model employed.

Comparison with related measurements of water dynamics in spatial confinement. Within PA membranes carefully hydrated by H_2O vapor, water diffusion differs markedly from bulk behavior, which remain Arrhenius down to the supercooled temperature range (Fig. 5b, main paper (Supplementary Table 1)). This behavior is analogous to that of water under confinement in hydrophilic spinodal Vycor glass of approximately 5 nm pore size, under full hydration conditions [12, 13]. When compared to Vycor, despite the difference in water uptake ($\simeq$ 14% $vs \simeq$ 25%, for PA and Vycor at 100% relative humidity [12, 14]), PA-confined water exhibits generally similar D_{tr} and jump length (l) and slightly lower τ_0 (Supplementary Table 1). However, the local water diffusion in PA is faster than that in Vycor ($D_{loc} =$ 2.6-3.4 and 1.4-2.5 10^{-5} cm^2s^{-1} at 260-270 K, respectively, Supplementary Table 1); this difference further increases for Vycor at lower hydration, and is thus likely caused by the comparatively hydrophilic character of glass.

Sample	T (K)	Technique	D_{loc}x10^{-5} (cm^2s^{-1})	D_{tr}x10^{-5} (cm^2s^{-1})	τ (ps)	l (Å)
PA_{vap}	250	$QENS_{TOF}$	1.9	0.53	3.9	1.1
$Vycor_{100\%}$ [12]	258	$QENS_{TOF}$	1.4	0.75	4.3	-
$Vycor_{52\%}$ [12]	258	$QENS_{TOF}$	1.1	1.19	16.0	-
$Vycor_{25\%}$ [12]	258	$QENS_{TOF}$	0.3	1.20	25.0	-
PA_{vap}	260	$QENS_{TOF}$	2.6	0.76	3.2	1.2
$Vycor_{100\%}$ [12]	268	$QENS_{TOF}$	2.5	1.01	2.7	-
$Vycor_{52\%}$ [12]	268	$QENS_{TOF}$	1.1	1.29	9.7	-
$Vycor_{25\%}$ [12]	268	$QENS_{TOF}$	0.4	1.36	20.0	-
PA_{vap}	270	$QENS_{TOF}$	3.4	0.98	2.4	1.2
PA_{vap}	270	$QENS_{BS}$	0.1	0.12	660	-
$Vycor_{100\%}$ [12]	278	$QENS_{TOF}$	3.3	1.40	2.3	-
$Vycor_{52\%}$ [12]	278	$QENS_{TOF}$	1.4	1.82	7.8	-
PA_{vap}	290	$QENS_{TOF}$	-	1.40	2.2	1.3
PA_{vap}	330	$QENS_{TOF}$	-	2.43	1.3	1.3
PA_{vap}	330	$QENS_{BS}$	-	0.23	460	-
PA_{liq}	250	$QENS_{TOF}$	-	0.43	20.5	2.3
PA_{liq}	260	$QENS_{TOF}$	-	0.84	4.9	1.6
PA [15]	268	$QENS_{TOF}$	-	1.06	4.2	-
PA_{liq}	270	$QENS_{TOF}$	-	1.08	3.2	1.4
PA [15]	280	$QENS_{TOF}$	-	1.31	1.8	-
PA_{liq}	290	$QENS_{TOF}$	-	1.92	1.5	1.3
PA [15]	290	$QENS_{TOF}$	-	1.69	1.6	-
PA [15]	300	$QENS_{TOF}$	-	1.98	1.0	-
PA [15]	325	$QENS_{TOF}$	-	4.09	1.0	-
PA_{liq}	330	$QENS_{TOF}$	-	4.80	-	-
Water [16]	240	pulsed-heating	-	0.13	-	-
Water [16]	245	pulsed-heating	-	0.23	-	-
Water [16]	250	pulsed-heating	-	0.38	-	-
Water [10]	253	$QENS_{TOF}$	-	0.38	22.7	2.4
Water [16]	255	pulsed-heating	-	0.50	-	-
Water [10]	256	$QENS_{TOF}$	-	0.48	10.8	1.8
Water [10]	258	$QENS_{TOF}$	-	0.56	8.9	1.7
Water [16]	260	pulsed-heating	-	0.75	-	-
Water [10]	261	$QENS_{TOF}$	-	0.63	7.6	1.7
Water [10]	263	$QENS_{TOF}$	-	0.70	6.5	1.6
Water [10]	268	$QENS_{TOF}$	-	0.85	4.7	1.5
Water [10]	278	$QENS_{TOF}$	-	1.35	2.3	1.3
Water [10]	285	$QENS_{TOF}$	-	1.80	1.7	1.2
Water [10]	293	$QENS_{TOF}$	-	2.40	1.2	1.3
Water [10]	313	$QENS_{TOF}$	-	3.20	0.9	0.9
Water [9]	330	NMR	-	4.8	-	-

Table 1: Summary of water dynamics parameters obtained for bulk water and with PA and Vycor confinement.

Comparison between water dynamics observed in H_2O vapor hydrated and immersed PA membranes. In order to evaluate the effect of vapor vs. liquid water hydration, and isolate confined and effectively 'bulk' water, we also investigated PA membranes immersed in liquid H_2O (Fig. 5f, main paper) and compared it with both D_2O and H_2O vapor hydrated PA crumpled polymer (Supplementary Fig. 13,15).

As shown in Supplementary Fig. 15, PA-membranes immersed in H_2O investigated across a broad temperature range exhibit three distinct regimes. Specifically, between 150-230 K only PA segmental relaxations are visible; above 230 K the scattering profile shows clear diffusive motions (i.e. with a Q-dependent Lorentzian), which we associate with confined water. Above approximately 280 K, this diffusive component can be associated with bulk-like water. These regimes are further confirmed by the DSC profile (Fig. 5g, main paper, light green curves), as well as by the comparison between the associated activation energies (Supplementary Fig. 16a). We compare our results with those reported in literature for both PA [17, 18, 15] and Vycor [12] confined and bulk water [10, 9] in Supplementary Fig. 16b-d, also included in Supplementary Table 1.

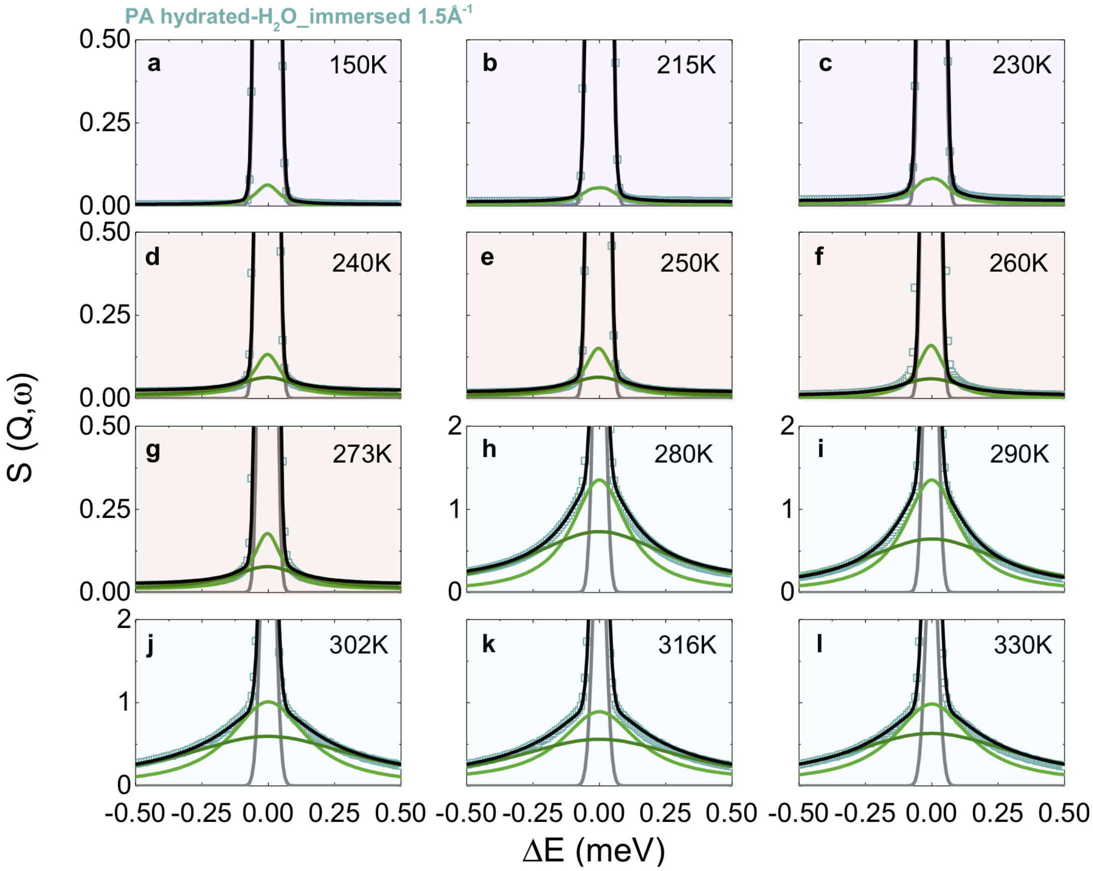

Supplementary Figure 15: Time of flight (IN5) data at Q = 1.5 Å^{-1} for H_2O immersed PA samples as function of temperature, from 150 to 330 K. Each panel shows S(Q,ω) with the global fit (black), the instrumental resolution (45 μeV, in grey), and the 1st, narrow Lorentzian function describing the polymer relaxation (light green) and the 2nd, broader Lorentzian function (dark green) describing the water dynamics. The 'purple' shading indicates that PA segmental relaxation is visible, while 'red' indicates that water diffusive motions (in confinement) are visible, and 'light green' indicates that a bulk-like water signal is also visible. Source data are available at DOI:10.5291/ILL-DATA.9-11-1809 and 10.5291/ILL-DATA.9-11-5311718.

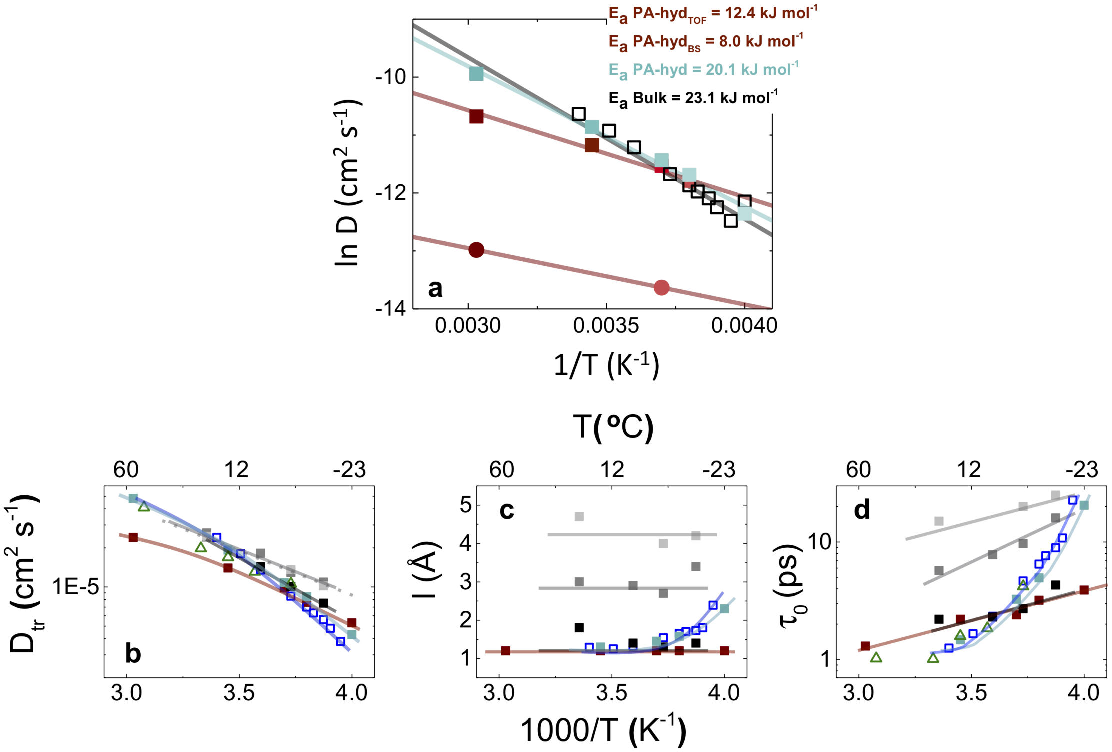

Supplementary Figure 16: a) Arrhenius behaviour for $D = D_0 exp(-E_a/kT)$ from whose slope is calculated the activation energy (E_a) associated to the water translational diffusion under the various conditions investigated. These include: H_2O-hydrated PA (D_{tr} and long-range D_{lr} diffusion, obtained from TOF and BS, respectively) and H_2O-immersed PA, compared to the value for bulk water [10]. b-d) Comparison between dynamic parameters obtained for H_2O-hydrated PA (dark red) and H_2O-immersed PA (light green), compared to previous work on immersed PA (green open markers) [17, 18, 15], for Vycor glass at different hydration levels (100% black, 52% dark grey, and 25% light grey markers) [12] and bulk water (blue open markers) [10, 9]. Panel (b) shows the temperature dependence of D_{tr}, while panel (c) shows the jump length ($D_{tr} = < l^2 >/6\tau$, and (d) shows the residence time τ_0, with lines serving as guide to the eye. Source data are provided as a Source Data file.

Supplementary Note 7. *Water uptake and proton fraction*

For clarity we provide detailed sample masses and hydration estimates to support the values used for mobile and immobile fractions, comparison between vapor-hydrated and immersed samples, as well as water uptake λ.

For hydrated PA in H_2O vapor, the mass of hydrated polymer = 0.77g = 0.60g dry polymer (= 0.0019mol) + 0.10g H_2O (= 0.0055mol); mass uptake = 14%wt, corresponding to water uptake (λ) = 3. For the hydrated PA in D_2O vapor, the mass of hydrated polymer = 0.71g = 0.60g dry polymer (= 0.0019mol) + 0.11g D_2O (= 0.0055mol), corresponding to mass uptake = 15%wt, and water uptake (λ) = 3. In order to compute the proton fraction in hydrated samples (fully hydrated in vapour), we write protons in the system = 14 (PA unit = 12 + water = 2), and thus Φ_W = (0.0019*12)/(0.0019*12+0.0055*2) = 0.33, Φ_P = (0.0055*2)/(0.0019*12+0.0055*2) = 0.67.

For the hydrated polymer (H_2O-immersed), we obtain mass of H_2O-immersed polymer = 0.60g = 0.30g dry polymer (= 0.0009mol) + 0.30g H_2O (= 0.017mol), and thus mass uptake = 50%wt and water uptake (λ) = 19. For the hydrated polymer (D_2O-immersed), we obtain mass of D_2O-immersed polymer = 0.60g = 0.30g dry polymer (= 0.0009mol) + 0.34g D_2O (= 0.017mol), and thus mass uptake = 54%wt, and water uptake (λ) = 19. Therefore, the proton fraction in hydrated samples (immersed in liquid water) is computed from the number of protons in the system = 14 (PA unit = 12 + water = 2), yielding Φ_W = (0.0009*12)/(0.009*12+0.017*2) = 0.76 and Φ_P = (0.017*2)/(0.009*12+0.017*2) = 0.24

Supplementary Note 8. *Relationship between multi-modal diffusion and membrane permeance*

Variations in the IP membrane fabrication (e.g. reactant relative and absolute concentration, reaction time, etc.) are well known to result in a wide range of performance [19, 20, 21, 22, 23], and formal property-performance relationships suitable for RO membrane design have not yet been established. Often, engineering performance is rationalised in terms of solution-diffusion type models [24] which do not explicitly account for well-known molecular and nanoscale heterogeneity (e.g. pore morphology and topology). Coronell and co-workers [19, 20, 21] have introduced descriptive strategies to account for these 'microscale variations' employing 'carefully weighting' of contributions based on some knowledge of morphology and connectivity [25]. We start from the 'macroscale model'

$$\frac{\frac{P}{\nu}}{\delta} = \frac{DK}{\delta}\frac{C_{bulk}\nu}{RT} \tag{S27}$$

where P ($m^2s^{-1}Pa^{-1}$) is the permeability of the active layer, δ (m) is the thickness, K (dimensionless) is the water partition, D (m^2s^{-1}) is the diffusion coefficients, C_{Bulk} is the concentration of bulk water (55.5 10^3 mol m^{-3}), ν is the molar volume of water (18.02 10^{-6} m^3mol^{-1}), R is the ideal gas constant (8.314 m^3Pa $K^{-1}mol^{-1}$) and T (K) is the absolute temperature. We then implement this model using:

1. experimentally calculated thickness, water uptake and void fraction f_{voids} computed according to [21]

$$f_{voids} = \frac{(m_l - m_v)\rho_{PA}}{m_{PA}\rho_w} \tag{S28}$$

   where m_{PA}, m_l and m_v (ng cm^{-2}) are the mass of the dry membrane, the water uptake when the membrane is exposed to liquid water and humidified nitrogen, respectively, and ρ_{PA} and ρ_w are the density of the dry membrane and water, respectively.

2. The water partition coefficient, K, under the assumption of absence of voids ($K_{polymer}$) as well as complete void interconnectivity (K_{voids}) [19, 20, 21]

$$K_{polymer} = \frac{(\frac{m_w}{MW_w\delta})}{C_{bulk}} \tag{S29}$$

$$K_{voids} = \frac{(\frac{m_w}{MW_w \delta (1-f_{void})})}{C_{bulk}} \tag{S30}$$

where MW_w (18.02 g mol^{-1}) and m_w (g) correspond to the molar mass of water and the mass of water sorbed by the active layer.

3. We then employ D_{tr} and D_{lr} to account of the diffusion within the active layer and within voids, respectively, since the local diffusion coefficient does not pertain to transport.

Within this framework, we compute a permeance (Supplementary Fig. 17) by weighing the two contributions (D_{tr} and D_{lr}) by their respective partition coefficient:

$$\frac{\frac{P}{\nu}}{\delta} = (1 - f_{voids})(\frac{D_{tr} K_{polymer}}{\delta} \frac{C_{bulk}\nu}{RT}) + (f_{voids})(\frac{D_{lr} K_{voids}}{\delta} \frac{C_{bulk} + \nu}{RT}) \tag{S31}$$

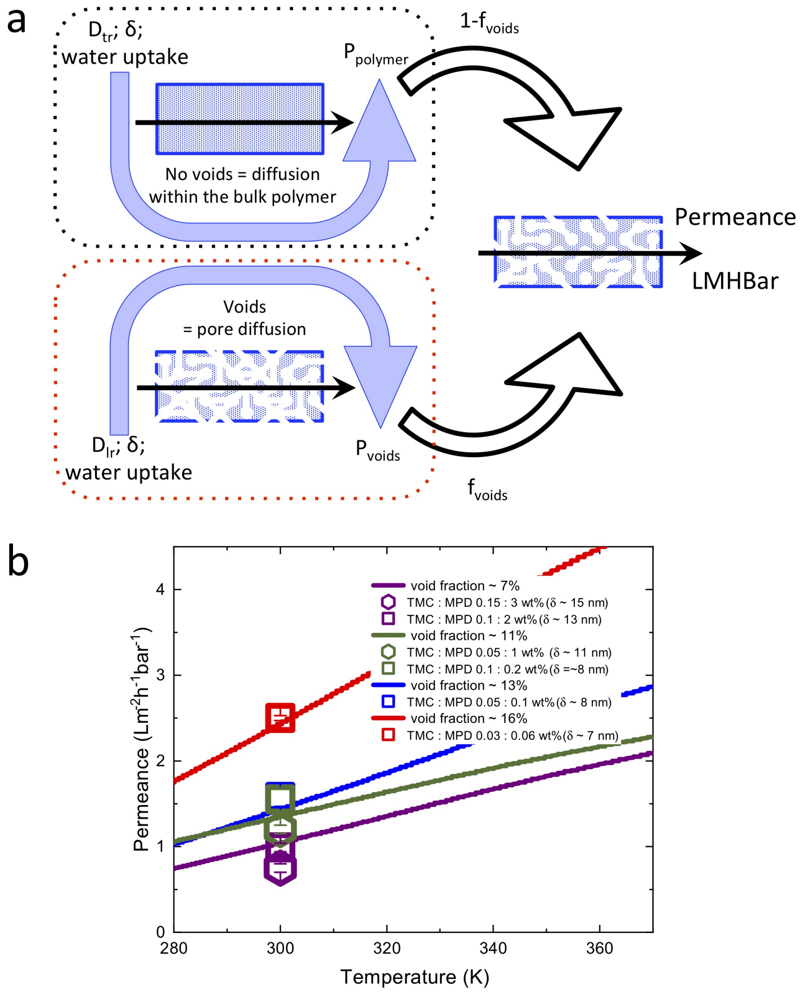

Supplementary Figure 17: a) Schematic of the approach used to compute membrane permeance from structural (thickness -δ-, water uptake and void fraction -f_{voids}-) and dynamical (D_{tr} and D_{lr}) parameters. b) Comparison between membrane permeance computed according to Eq. (S31) and macroscopic data for PA membranes prepared using similar synthetic approach (e.g. 1 min reaction time [26]). The legend includes the film thickness and void fraction employed in the calculation, as well as membrane stoichiometry. Source data are provided as a Source Data file.

Such models are, however, very sensitive to 'void fraction' (and related uptake) as well as thickness, which can be complex to measure exactly in heterogeneous media; for this reason, we attempt to utilise the same diffusion coefficients as *forward* prediction of the macroscopic performance of diverse membranes of similar chemical structure. Supplementary Fig. 17 shows the measured and calculated permeances with void fractions ranging from 7 to 16% obtained from QCM and AFM (for dry and hydrated mass, and effective thickness). We note that neglecting D_{lr} (i.e. setting $D_{lr} = 0$) in Eq. (S31), keeping the remaining parameters constant, yields a considerably smaller permeance, which indicates its significant impact on performance within the framework of the model.

The presence of potentially active and inactive, as well as connected and disjoined pathways within PA nanostructure makes estimating permeability very challenging. For instance, membrane dehydration has been shown to causes a non-fully reversible roughness deformation which has been related to a reduction of permeability [27]. Employing neutron reflectivity (NR) on thin-film membranes (of equivalent reaction stoichiometry) as a function of RH, we have simultaneously resolved membrane swelling and water uptake, as shown in Supplementary Fig. 18 [23]. The data appear to be fully reversible; furthermore, upon reaching 100% RH, we do not see evidence of further swelling or water uptake over time, suggesting that capillary condensation has a comparatively small impact. Further, we have found that although hydration has a marginal effect in polymer dynamics, it affects the fraction of active segments at a fixed temperature. Therefore, we expect that factors that modulate the PA (nanoscale) morphology, such as hydration, pressure, and conditioning agents will impact the macroscopic performance of membranes and thus the weighing of the various diffusion coefficients.

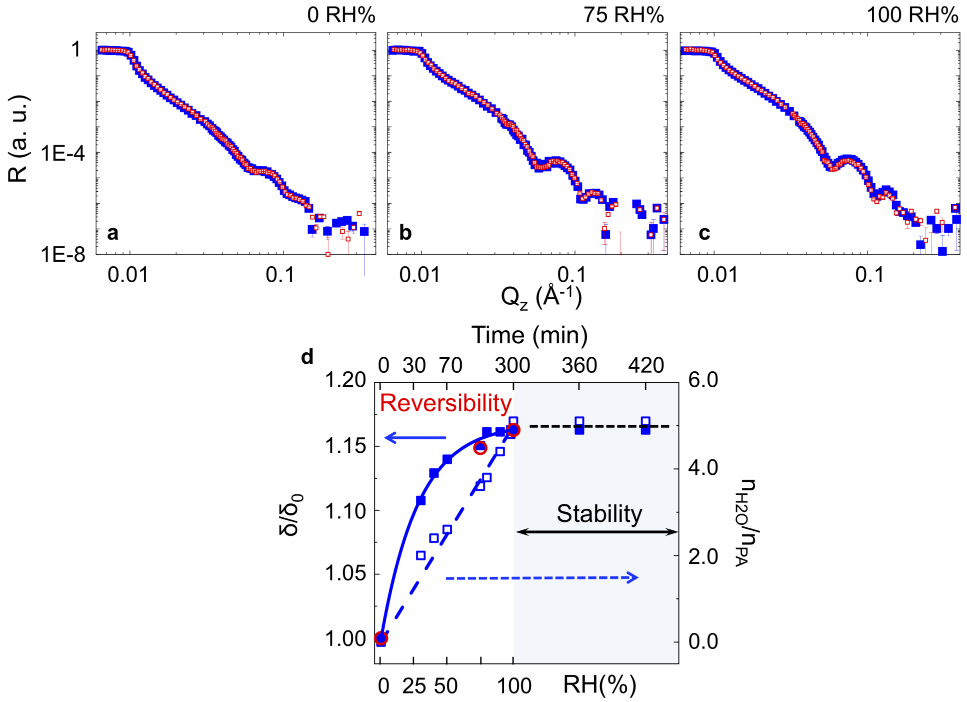

Supplementary Figure 18: a-c) NR profiles obtained at three RH% (0, 75 and 100 RH% [23]). The data show the reversibility of the membrane hydration/dehydration (blue data points for hydration, and red data points for dehydration) process. d) Variation of water uptake (n_{H2O}/n_{PA}) and membrane swelling (δ/δ_0) as function of relative humidity for TMC/MPD thin film, at 10 min reaction time, for TMC/MPD 0.005/0.1 wt%. In the plot is also reported the time scale to show the stability of the film. Source data are provided as a Source Data file.

References

[1] LAMP, the Large Array Manipulation Program, 1996.

[2] B. Frick, J. Combet, and L. van Eijck. New possibilities with inelastic fixed window scans and linear motor doppler drives on high resolution neutron backscattering spectrometers. *Nuclear Instruments and Methods in Physics Research Section A: Accelerators, Spectrometers, Detectors and Associated Equipment*, 669:7 – 13, 2012.

[3] Marc Bée. *Quasielastic Neutron Scattering*. Adam Hilger, Bristol, 1988.

[4] A. Pajzderska, M. A. Gonzalez, and J. Wasicki. Quasielastic neutron scattering study of pyridinium cation reorientation in thiourea pyridinium nitrate inclusion compound. *The Journal of Chemical Physics*, 128(8):084507, 2008.

[5] J. Muscatello, E.A. Müller, A.A. Mostofi, and A.P. Sutton. Multiscale molecular simulations of the formation and structure of polyamide membranes created by interfacial polymerization. *Journal of Membrane Science*, 527:180 – 190, 2017.

[6] Susumu Ikeda and Kazuo Matsuda. The activation energy spectrum of nylon 6 in the γ relaxation region obtained from depolarization current. *Japanese Journal of Applied Physics*, 15(6):963, 1976.

[7] Bart Vorselaars, Alexey V. Lyulin, and M. A. J. Michels. Development of heterogeneity near the glass transition:â phenyl-ring-flip motions in polystyrene. *Macromolecules*, 40(16):6001–6011, 2007.

[8] Rajesh Khare and Michael E. Paulaitis. Molecular simulations of cooperative ring flip motions in single chains of polystyrene. *Chemical Engineering Science*, 49(17):2867 – 2879, 1994.

[9] F. X. Prielmeier, E. W. Lang, R. J. Speedy, and H.-D. Lüdemann. The pressure dependence of self diffusion in supercooled light and heavy water. *Berichte der Bunsengesellschaft für physikalische Chemie*, 92(10):1111 – 1117, 1988.

[10] J. Teixeira, M.-C. Bellissent-Funel, S. H. Chen, and A. J. Dianoux. Experimental determination of the nature of diffusive motions of water molecules at low temperatures. *Phys. Rev. A*, 31:1913 – 1917, Mar 1985.

[11] F. Volino and A.J. Dianoux. Neutron incoherent scattering law for diffusion in a potential of spherical symmetry: general formalism and application to diffusion inside a sphere. *Molecular Physics*, 41(2):271–279, 1980.

[12] M.-C. Bellissent-Funel, S. H. Chen, and J.-M. Zanotti. Single-particle dynamics of water molecules in confined space. *Phys. Rev. E*, 51:4558–4569, May 1995.

[13] J.-M. Zanotti, M.-C. Bellissent-Funel, and S.-H. Chen. Relaxational dynamics of supercooled water in porous glass. *Phys. Rev. E*, 59:3084 – 3093, Mar 1999.

[14] Marie-Claire Bellissent-Funel. Structure and dynamics of water near hydrophilic surfaces. *Journal of Molecular Liquids*, 78(1):19 – 28, 1998. Proceedings of the 25th International Conference on Solution Chemistry.

[15] V.K. Sharma, S. Mitra, P. Singh, F. Jurányi, and R. Mukhopadhyay. Diffusion of water in nano-porous polyamide membranes: Quasielastic neutron scattering study. *The European Physical Journal Special Topics*, 189(1):217 – 221, Oct 2010.

[16] Yuntao Xu, Nikolay G. Petrik, R. Scott Smith, Bruce D. Kay, and Greg A. Kimmel. Growth rate of crystalline ice and the diffusivity of supercooled water from 126 to 262 k. *Proceedings of the National Academy of Sciences*, 113(52):14921–14925, 2016.

[17] V.K. Sharma, P.S. Singh, S. Gautam, P. Maheshwari, D. Dutta, and R. Mukhopadhyay. Dynamics of water sorbed in reverse osmosis polyamide membrane. *Journal of Membrane Science*, 326(2):667 – 671, 2009.

[18] V.K. Sharma, P.S. Singh, S. Gautam, S. Mitra, and R. Mukhopadhyay. Diffusion of water in nanoporous nf polyamide membrane. *Chemical Physics Letters*, 478(1):56 – 60, 2009.

[19] Lin Lin, Timothy M. Weigand, Matthew W. Farthing, Panitan Jutaporn, Cass T. Miller, and Orlando Coronell. Relative importance of geometrical and intrinsic water transport properties of active layers in the water permeability of polyamide thin-film composite membranes. *Journal of Membrane Science*, 564:935 – 944, 2018.

[20] Lamar A. Perry and Orlando Coronell. Reliable, bench-top measurements of charge density in the active layers of thin-film composite and nanocomposite membranes using quartz crystal microbalance technology. *Journal of Membrane Science*, 429:23 – 33, 2013.

[21] Lin Lin, Rene Lopez, Guy Z. Ramon, and Orlando Coronell. Investigating the void structure of the polyamide active layers of thin-film composite membranes. *Journal of Membrane Science*, 497:365 – 376, 2016.

[22] Zhiwei Jiang, Santanu Karan, and Andrew G. Livingston. Water transport through ultrathin polyamide nanofilms used for reverse osmosis. *Advanced Materials*, 30(15):1705973, 2018.

[23] Fabrizia Foglia, Santanu Karan, Manuela Nania, Zhiwei Jiang, Alexandra E. Porter, Robert Barker, Andrew G. Livingston, and João T. Cabral. Neutron reflectivity and performance of polyamide nanofilms for water desalination. *Advanced Functional Materials*, 27(37):1701738–n/a, 2017. 1701738.

[24] J.G. Wijmans and R.W. Baker. The solution-diffusion model: a review. *Journal of Membrane Science*, 107(1):1 – 21, 1995.

[25] Fabrizia Foglia, Adam J. Clancy, Jasper Berry-Gair, Karolina Lisowska, Martin C. Wilding, Theo M. Suter, Thomas S. Miller, Keenan Smith, Franz Demmel, Markus Appel, Victoria García Sakai, Andrea Sella, Christopher A. Howard, Madhusudan Tyagi, Furio Corà, and Paul F. McMillan. Aquaporin-like water transport in nanoporous crystalline layered carbon nitride. *Science Advances*, 6(39), 2020.

[26] Zhiwei Jiang, Santanu Karan, and Andrew G. Livingston. Water transport through ultrathin polyamide nanofilms used for reverse osmosis. *Advanced Materials*, 30(15):1705973, 2018.

[27] Xiaoxiao Song, Bowen Gan, Zhe Yang, Chuyang Y. Tang, and Congjie Gao. Confined nanobubbles shape the surface roughness structures of thin film composite polyamide desalination membranes. *Journal of Membrane Science*, 582:342 – 349, 2019.
